# Eye-Tracking Research: A Bibliometric Analysis Based on Scopus

**DOI:** 10.3390/jemr19050103

**Published:** 2026-09-16

**Authors:** Mehmet Fatih Özalp, Hicran Hanım Halaç, Nuray Özkaraca Özalp, Fikret Bademci

**Affiliations:** 1 Independent Researcher, Düzce 81620, Turkey; fozalp79@gmail.com; 2 Department of Architecture, Faculty of Architecture, Eskisehir Technical University, Eskişehir 26555, Turkey; hhhalac@eskisehir.edu.tr; 3 Department of Architecture, Faculty of Fine Arts and Design, Siirt University, Siirt 56000, Turkey; nuray.ozkaraca@siirt.edu.tr; 4 School of Architecture, University of Liverpool, Liverpool L69 7ZG, UK

**Keywords:** eye-tracking, gaze tracking, bibliometric analysis, research trends

## Abstract

Eye tracking is an interdisciplinary research method used in many fields, including engineering, architecture, health sciences, psychology, marketing, and human–computer interaction. Although it is often perceived as a modern research method, its origins can be traced back to the nineteenth century. This study aims to identify the developmental trends and thematic structure of eye-tracking research from its early history to the present. Bibliometric analysis was used to evaluate studies in the field of eye-tracking. A total of 45,230 publications identified in the Scopus database using eye-tracking search terms were analyzed. The most productive authors, institutions, countries, publication sources, keywords, and thematic trends were examined using Bibliometrix in the RStudio environment. The findings show substantial growth in eye-tracking research during 2020–2025, with 22,130 publications recorded during this period. The analysis also shows that the share of eye-tracking publications in total Scopus output increased from 0.0134% in 2000 to 0.0963% in 2025, while the search sensitivity analysis demonstrates that terminology choices substantially affect the size and composition of the retrieved corpus. Computer Science accounted for the largest share of publications. The United States had the highest number of publications. Based on Scopus affiliation records, the University of California system had the highest publication count among the institutional structures identified in the dataset, although this figure may reflect the combined representation of different campuses and affiliated units and should therefore not be interpreted as a direct comparison of individual campuses. In addition to fundamental concepts such as eye movements and visual attention, themes such as EEG, virtual reality, augmented reality, computer vision, machine learning, and deep learning have become more prominent in recent years. Overall, the findings reveal academic orientations and temporal trends in eye-tracking research. They also provide a framework for potential future research directions.

## 1. Introduction

Although eye-tracking technology is now widely regarded as a modern research method, its origins date back to the nineteenth century, when systematic investigations of the reading process first began [1]. Initially, researchers examined eye movements through direct observation without the aid of dedicated recording equipment. In these early studies, the researcher stood behind the participant and indirectly observed eye movements through a mirror positioned on the pages of the book being read. Although this method was unable to provide quantitative or highly precise measurements, it led to important pioneering discoveries regarding the mechanisms of visual perception during reading [2]. The identity of the first researcher to investigate eye movements during reading remains a matter of debate. However, most historical accounts identify the French ophthalmologist Louis Émile Javal as the first to describe eye movements during reading in the late 1870s, a period widely regarded as marking the beginning of eye-tracking research. In 1879, Javal showed that the eyes do not move continuously along a line of text during reading, as previously assumed. Instead, reading is not a linear process but consists of rapid eye movements (saccades) and brief pauses at specific points (fixations) [3]. In addition, recent historical studies suggest that the traditional account of Javal should be considered with greater caution. In 1879, Javal used the term “saccade” to describe eye movements. However, studies measuring eye movements during reading were conducted by Lamare in Javal’s laboratory. In the same year, Hering used a similar technique to describe the discontinuous nature of eye movements during reading [4].

The systematic study of eye movements was conducted by E. Huey in 1898. Huey also developed one of the earliest eye-tracking devices, which incorporated an iris aperture connected to an aluminum pointer. However, the device was highly invasive and caused considerable discomfort to participants. During a similar period, E. B. Delabarre also developed a mechanical eye-tracking method using a plaster cap that adhered to the moist surface of the eye. Although these techniques represented important methodological advances, both posed significant limitations with respect to participant comfort and eye health [2]. A major milestone in the development of eye-tracking technology was the introduction of the first non-invasive optical eye tracker by R. Dodge and T. S. Cline in 1901. The device could record only horizontal eye movements and required head stabilization, which were important limitations. Nevertheless, it contributed to the development of the field by showing that visual information is acquired discontinuously during eye movements [2,3]. Further technological advances followed in 1905, when Ch. H. Judd, C. N. McAllister, and W. M. Steel developed an optical device capable of recording both horizontal and vertical eye movements without mechanical contact with the eye. The introduction of film recording techniques in the 1920s enabled the development of non-invasive methods capable of recording eye movements in two directions. During the same period, electrical eye-tracking techniques also emerged, based on measuring changes in the eye’s electrical potential using electrodes. In 1922, E. Schott improved the accuracy and reliability of eye movement measurements using the EOG technique. This period marked the second major phase in the evolution of eye-tracking research, driven by rapid technological advances and the growing influence of behavioral psychology [5].

**Figure 1:**
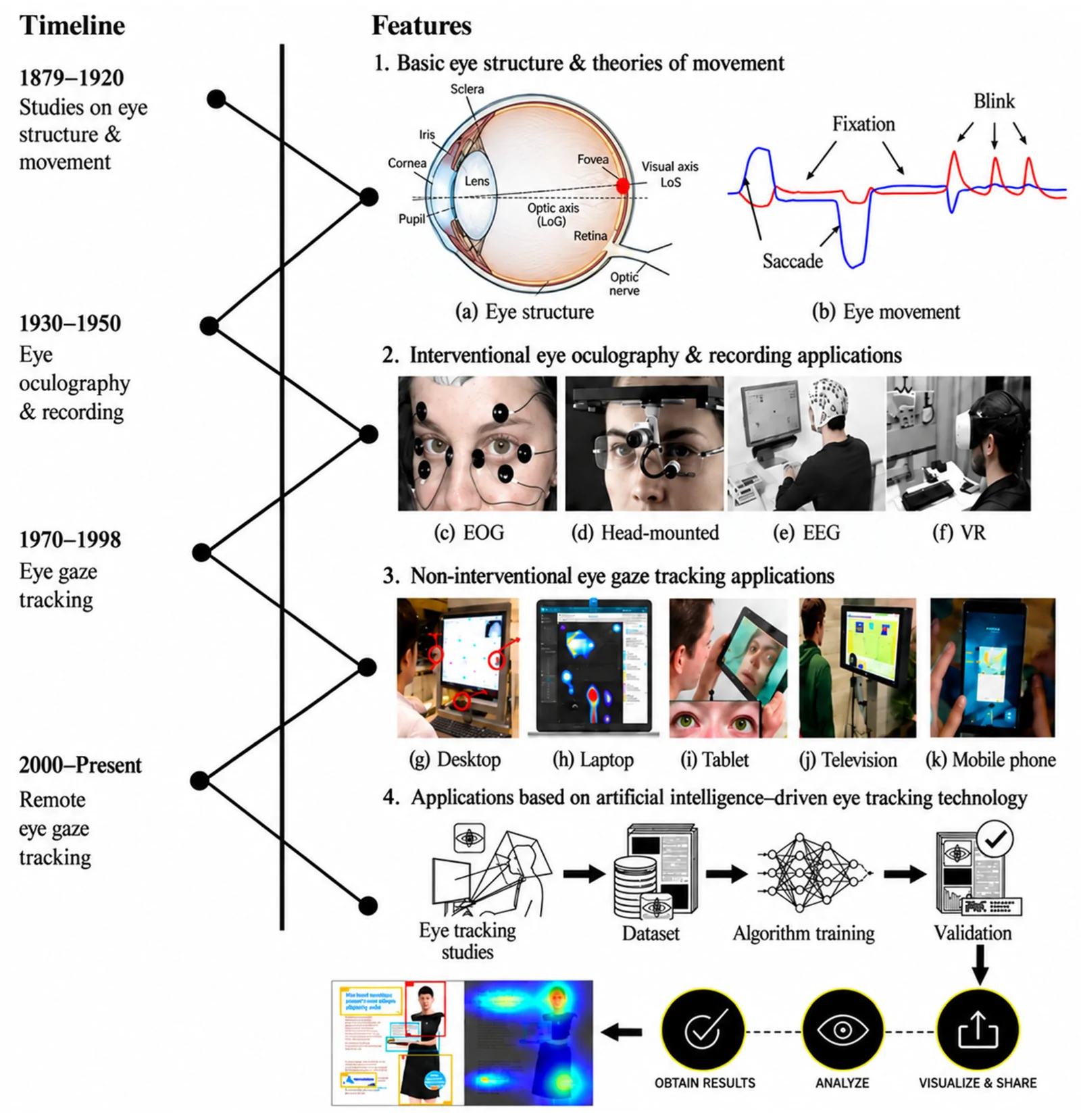
Historical development of eye-tracking research. Translated and reproduced from Ref. [8].

Eye-tracking technology advanced substantially during the 1930s. Guy Thomas Buswell developed a non-contact optical system that recorded the two-dimensional movements of a single eye by analyzing light reflected from the cornea. In 1935, Buswell recorded eye movements on film for the first time. In experiments involving 200 participants viewing pictures, he obtained approximately 2000 eye-movement records, each consisting of numerous fixations [6]. These studies represented one of the earliest large-scale investigations of visual attention [7]. In the 1940s, P. M. Fitts and his colleagues extended eye tracking to applied research using film-based recording systems. In 1948, Hamilton Hartridge and Landsborough C. Thomson introduced the first head-mounted optical eye tracker [2] (Figure 1). This device improved measurement accuracy and reduced constraints associated with head movements.

During the 1950s and 1960s, eye-tracking systems became increasingly mobile, compact, and user-friendly. Moreover, the simultaneous scene-and-gaze recording approach proposed by J. F. Mackworth and N. H. Mackworth facilitated the interpretation of eye movement data and contributed to the development of modern eye-tracking systems [2]. In the 1980s, computer technology advanced to the point where real-time eye tracking became possible. This development enabled the use of video-based eye-tracking systems in human–computer interaction. With the widespread adoption of these systems in the 1990s, access to eye-tracking technology increased, and its use expanded across various research fields. Initially used to evaluate advertisements in print magazines, eye-tracking applications were integrated into studies of user experience on the internet in the late 1990s, coinciding with the widespread adoption of online product presentations [9]. Starting in the 2000s, advances in eye-tracking technology expanded the scope of its applications. Eye tracking has since become widely used in scientific and commercial fields. It is used in the study of reading and learning processes and the analysis of consumer behavior. It is also used in the diagnosis and treatment of cognitive and neurological disorders and in the development of alternative communication options for people with disabilities [10]. In 2001, Tobii made a significant contribution to eye-tracking research by introducing the first remote eye-tracking device. Tobii develops eye-tracking systems based on camera and near-infrared sensor technologies. These systems measure head and eye movements and use advanced signal processing and artificial intelligence methods to determine the point of gaze and analyze attention distribution [11,12]. In parallel with these developments, eye-tracking technologies have become more accessible and have been increasingly adopted by researchers across different disciplines [13]. In recent years, eye-tracking studies based on predictive algorithms have also been conducted without the use of human participants. These studies have emerged alongside advances in machine learning and artificial intelligence (Figure 1).

Today, eye-tracking technologies include hardware-based eye-tracking systems (Tobii Pro [14], EyeLink [15], etc.—fixed, screen-based systems—and Tobii Glasses [14], Pupil Labs [16], etc.—wearable, mobile systems), webcam-based eye-tracking systems (GazeRecorder [17], EyeSee [18], RealEye [19], etc.), artificial intelligence-based (predictive) eye-tracking systems (Attention Insight [20], VisualEyes [21], EyeQuant [22], 3M-VAS [23], etc.), and hybrid systems. Eye tracking has evolved into an interdisciplinary research method used across a wide range of fields, including medicine, psychology, architecture, design, marketing, and human–computer interaction. As technological developments have made eye-tracking technologies more accessible, they have been increasingly adopted by researchers from different disciplines. This study aims to identify the developmental trends and thematic structure of eye-tracking research from the past to the present.

An examination of existing bibliometric studies in the field of eye tracking reveals that most research is limited to specific time periods, databases, or thematic subfields. For example, in a study conducted by Korkmaz (2026), 1033 publications released between 2020 and 2025 in the Web of Science (WoS) and Scopus databases were analyzed using the VOSviewer software [24]. Similarly, Lai, Su and She (2025) examined 374 articles published in 19 journals in the Web of Science database between 2001 and 2024 using the Bibliometrix and CiteSpace tools [25]. Furthermore, Cherukat and Shabnam (2026) evaluated 6846 articles published in the Scopus database between 1955 and 2025 using the R Bibliometrix package and VOSviewer [26]. However, a substantial proportion of bibliometric studies in the field of eye tracking have focused on specific thematic areas, such as marketing [27], consumer behavior [28], virtual reality [29], and tourism [30]. As presented comparatively in Table 1, most existing bibliometric studies focus on specific thematic areas, limited time periods, or smaller datasets. In contrast, large-scale bibliometric studies covering multiple disciplines without imposing a time restriction remain limited. This makes it difficult to assess the historical development, interdisciplinary diffusion, and thematic transformation of eye-tracking research in an integrated manner. More specifically, Korkmaz (2026) [24] examined eye-tracking research in the context of human–computer interaction, while Lai, Su and She (2025) [25] focused on educational technology, and Salgado-Fernández et al. (2022) [31], examined the relationship between eye movements and academic performance using bibliometric and citation network analysis. The present study differs from these works by examining eye-tracking research across multiple disciplines and by additionally evaluating the share of eye-tracking publications within overall Scopus output and the sensitivity of the retrieved corpus to alternative search terminology.

Building on this research gap, this study analyzes 45,230 publications indexed in the Scopus database using bibliometric methods. The study provides an integrated assessment of the historical development, scientific production structure, collaboration networks, and thematic trends of eye-tracking research. Unlike previous bibliometric studies, it applies no time restriction and uses a large-scale dataset covering multiple disciplines. In addition to examining publication, collaboration, and thematic patterns, the study evaluates the share of eye-tracking publications relative to overall Scopus output over time and examines how changes in search terminology affect the size and composition of the retrieved corpus. These analyses provide additional insight into the growth of eye-tracking research and the sensitivity of bibliometric findings to search terminology. It aims to contribute to the existing body of knowledge in the field and provide a general framework for future research.

## 2. Materials and Methods

The term “bibliometrics” was first defined in 1969 by Alan Pritchard as “the application of mathematical and statistical methods to books and other means of information transmission.” Bibliometrics aims to examine publication patterns in a specific field through the quantitative analysis of empirical data from the published literature [42]. In this context, bibliometric methods enable researchers to systematically evaluate the literature in their fields of study and identify key themes that emerge in the literature [43,44]. Bibliometric analysis is frequently used in conjunction with science mapping techniques to reveal and visualize the scientific structure of a specific research field [45,46]. In this study, the bibliometric analysis method was employed to uncover the developmental trends and thematic structure of eye tracking from the past to the present and to contribute to future research. The bibliometric analysis was conducted using a two-stage methodological approach consisting of data collection and analysis.

Scopus was used as the primary bibliographic database for data collection [47]. The search was conducted using the TITLE-ABS-KEY field, which encompasses the title, abstract, and keyword fields in the Scopus database.

The complete search query was: TITLE-ABS-KEY (“eye tracking” OR “eye-tracking” OR “gaze tracking” OR “gaze behavior”).

The search was conducted on 20 January 2026 and yielded a total of 45,230 bibliographic records. No restrictions were imposed with respect to document type (e.g., journal articles, books, book chapters, and conference papers), subject area, publication language, publication year, or quartile. Using the same search criteria in the Web of Science (WoS) database yielded 30,461 publications. Scopus was selected primarily because the search strategy resulted in a larger dataset than that obtained from WoS and because Scopus provides data export options suitable for large-scale bibliometric analyses. In addition, Scopus allows the export of up to 20,000 records per export, whereas WoS has a limit of 500 records per export [48]. This difference presents practical and operational challenges when managing datasets of the size required for the present study. Based on these considerations, Scopus was selected as the data source for the study, and all bibliometric analyses were conducted using the 45,230 bibliographic records retrieved from this database. Nevertheless, the exclusive use of Scopus inevitably limits the scope of the study to publications indexed in this database. Consequently, publications indexed in Web of Science, Dimensions, or other bibliographic databases but not covered by Scopus were not included in the analysis. Therefore, the findings should be interpreted within the scope of the literature indexed by Scopus. Comparative analyses incorporating multiple bibliographic databases are recommended for future research to provide a broader and more comprehensive representation of the literature.

**Figure 2:**
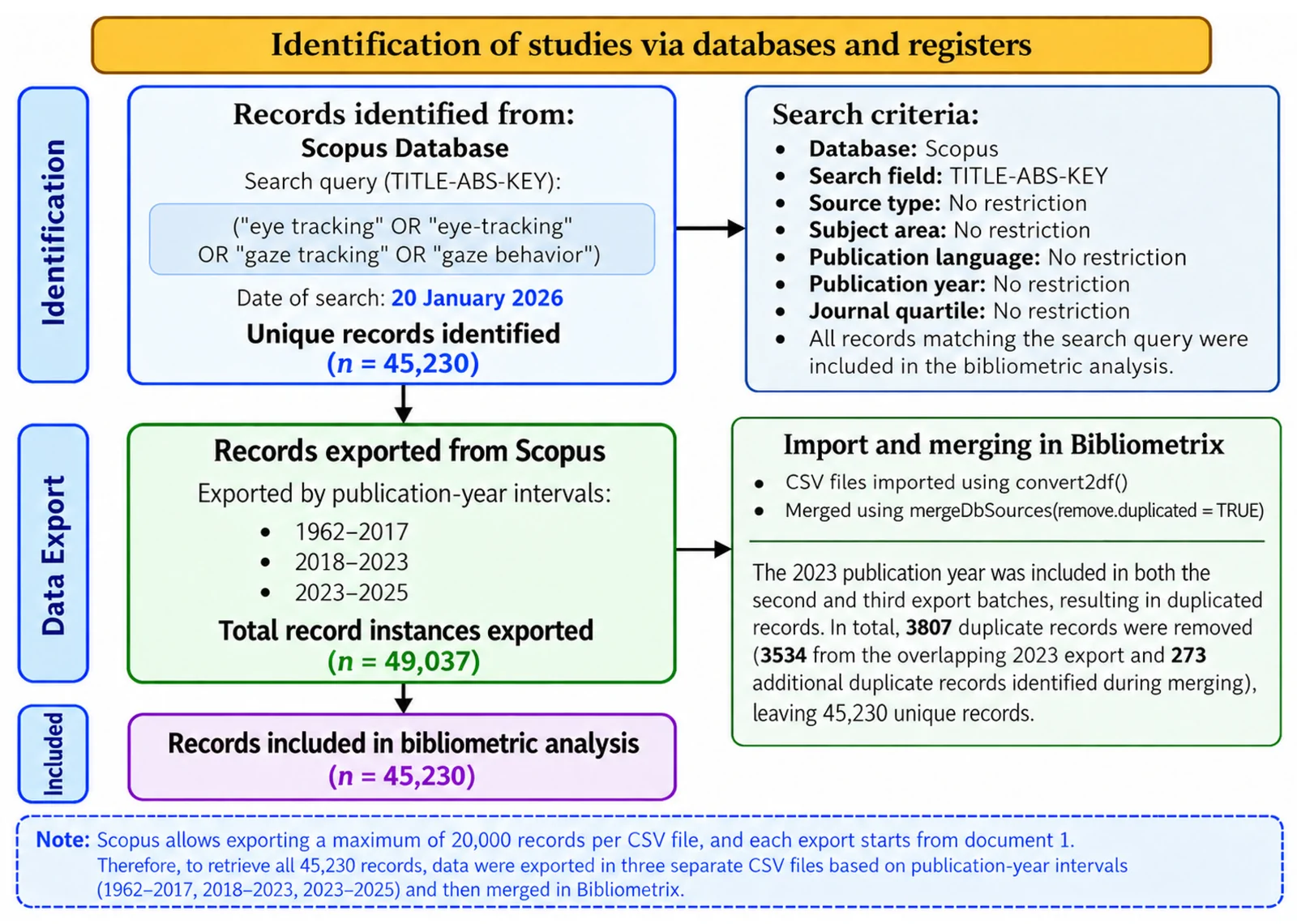
PRISMA-based flow diagram of the bibliometric data collection and analysis process (Scopus; 45,230 records included in the final analysis; 1962–2025).

During the export of records from the Scopus database in CSV format, a maximum of 20,000 records could be exported in a single operation. Therefore, the dataset was exported in three batches according to publication-year ranges: 1962–2017, 2018–2023, and 2023–2025. The overlap of the 2023 publication year between the second and third export batches resulted in repeated records. The three CSV files were imported using the convert2df() function of the Bibliometrix (v5.2.1) package and merged using the mergeDbSources() function with duplicate removal enabled. In total, 3807 duplicated records (3534 from the overlapping 2023 export and 273 additional duplicate records identified during the merging process) were removed, resulting in a final corpus of 45,230 records. All subsequent analyses were performed on the final dataset of 45,230 records (Figure 2).

During the data analysis phase, the Bibliometrix (v5.2.1) and Biblioshiny packages running in the RStudio v2026.01.0+392 (Posit Software, Boston, MA, USA) environment were used. The R-based Biblioshiny package, developed by Aria and Cuccurullo (2017) [49], enables more detailed analysis and visualization of data thanks to its structure, which incorporates advanced mathematical and statistical algorithms compared to other bibliometric analysis tools. Within the scope of this study, the R-based Biblioshiny package was used to analyze the research questions listed below:

- What is the distribution of studies in the field of eye tracking by year?
- What is the distribution of eye tracking-related studies by publication type?
- Which countries, institutions, and authors are the most productive in the field of eye tracking, and what are their collaboration patterns?
- What is the distribution of single- and multi-author studies in the field of eye tracking?
- What is the tripartite relationship among leading countries, authors, and keywords in the field of eye tracking?
- What are the most frequently used keywords in the field of eye tracking?
- What are the trending topics in the field of eye tracking?

In accordance with the research questions, standard bibliometric analysis functions available in the Bibliometrix/Biblioshiny (v5.2.1) package were used to perform analyses of annual scientific production, publication types, country, institutional, author, and source productivity, citation patterns, country and institutional collaboration networks, three-field plot analysis showing relationships among countries, authors, and keywords, the most frequently used keywords (Most Relevant Words), and trend topics. Network-based bibliometric analyses were conducted using the default network parameters available in Biblioshiny. The Association method was used to normalize link strength in country and institutional collaboration networks; clusters were identified using the Louvain clustering algorithm, and the Automatic Layout was selected for network visualization. In the Collaboration Network analyses, the network size was set to 50 nodes (Number of Nodes = 50), the repulsion force was set to 0.5 (Repulsion Force = 0.5), and the minimum number of edges was set to 1 (Minimum Number of Edges = 1); isolated nodes were excluded from the analyses (Remove Isolated Nodes = Yes). For the world collaboration maps, only links representing at least two co-publications were displayed (Minimum Edges = 2). In the three-field plot analysis, authors (Authors) were selected as the middle field, countries (Countries) as the left field, and keywords (Keywords) as the right field. The top 10 items with the highest productivity or frequency of use were visualized in each field (Number of Items = 10). Thematic mapping was conducted using author keywords (Author’s Keywords). A minimum cluster frequency threshold of 5 was applied, 250 keywords were considered, and the Louvain algorithm was used to generate the clusters.

### 2.1. Validation of the Dataset

To assess the possibility of including out-of-scope records in the search strategy, a validation study was conducted on the final dataset comprising 45,230 records used in the analyses. For this purpose, 300 records were selected from the final dataset using simple random sampling. To ensure the reproducibility of the sample, set.seed() was used in R (v4.5.2), and the records were selected without replacement using the sample() function (replace = FALSE).

The selected records were evaluated and classified as either within-scope or out-of-scope based on whether they were directly or meaningfully related to eye-tracking research. The assessment considered whether content related to eye tracking, gaze, and eye movements was present in the titles and abstracts as a research topic or research component. Where necessary, the publication content and bibliographic information accessible through the DOI were also examined.

Of the 300 records assessed, 257 (85.67%) were classified as within-scope and 43 (14.33%) as out-of-scope. Accordingly, the estimated proportion of out-of-scope records based on the validation sample was 14.33%, with a 95% Wilson confidence interval of 10.82–18.75% [50]. These findings indicate that the majority of the dataset (85.67%) is directly or meaningfully related to eye-tracking research, while also suggesting that the search strategy may include a certain proportion of out-of-scope records.

### 2.2. Evaluation of the Scope of the Search Strategy

The search strategy used in this study included four primary terms that directly represent the eye-tracking literature: “eye tracking,” “eye-tracking,” “gaze tracking,” and “gaze behavior.” The search query was applied to the TITLE-ABS-KEY fields in the Scopus database and was conducted on 20 January 2026. This query yielded 45,230 records. The earliest publication in the dataset dates back to 1962. Therefore, the bibliometric findings of this study are limited to Scopus-indexed publications from 1962 onward. The historical development of eye-tracking research before 1962 was not derived from the bibliometric dataset but was addressed as part of the historical background based on relevant secondary literature.

The four primary terms used in the search strategy were selected based on terminology that directly represents eye tracking and gaze behavior. “Eye tracking” and “eye-tracking” represent different spelling forms of the same concept, “gaze tracking” refers to the tracking of eye gaze, and “gaze behavior” refers to gaze behavior.

To assess the effect of the terminology used in the search strategy on the scope of the dataset, an additional retrieval-level sensitivity check was conducted. In this analysis, alternative and broader terminology related to eye movements was progressively added to the query. First, the British English term “gaze behaviour” was added to the query, resulting in no change in the number of records (45,230 records). The unchanged result may be explained by Scopus’s handling of British and American spelling variants within the TITLE-ABS-KEY field, where such variants are treated as equivalent. When the terms “eye movement” and “eye movements” were added, the number of records increased to 143,686. Finally, the addition of the terms “saccade,” “fixation,” “scanpath,” “oculomotor,” “pupillometry,” and “electrooculography” increased the total number of records to 553,231.

This comparison shows that the terminology used in the search strategy has a substantial effect on the scope and size of the dataset. Accordingly, it is acknowledged that the current search strategy does not capture all studies that use independent terminological variations related to eye movements. Therefore, the scope of the study is appropriately limited to the terminology included in the search strategy.

## 3. Results

Between 1962 and 2025, the Scopus dataset analyzed in this study comprised 45,230 publications distributed across 5273 different sources, including journals, books, and other publication types. The annual growth rate for the 1962–2025 period was calculated as 14.32%; however, because only one publication was recorded in 1962, this rate is highly sensitive to the initial baseline and should therefore be interpreted with caution. Accordingly, this rate was not considered a standalone indicator of the field’s relative growth. To control for the effect of the overall increase in publications indexed in Scopus, the annual output of eye-tracking publications was normalized against the total number of documents indexed in Scopus each year. The results show that the share of eye-tracking research within Scopus increased from 0.0005% in 1962 to 0.0134% in 2000, 0.0308% in 2010, and 0.0842% in 2020. Although this proportion declined to 0.0769% in 2022, it increased again in subsequent years, reaching 0.0963% in 2025. These results suggest that the increase in publications in the analyzed dataset was not solely attributable to the overall growth of the Scopus database and that the share of eye-tracking publications within the total Scopus literature increased over time.

A total of 83,655 authors contributed to the literature, of whom 2096 produced single-authored publications. Only 3420 publications were single-authored, suggesting a predominantly collaborative publication pattern in the analyzed dataset. The mean number of co-authors per document was 4.07, while the proportion of international co-authorship was 23.02%, further indicating the important role of collaboration in the eye-tracking literature.

When document types were examined, the 45,230 records in the dataset were found to be distributed across different publication types. Most of the publications were articles (28,215; 62.38%) and conference papers (13,202; 29.19%). These were followed by review articles (1220; 2.70%), book chapters (1123; 2.48%), and conference reviews (678; 1.50%). The dataset also included records classified as books (88; 0.19%), data papers (55; 0.12%), editorials (173; 0.38%), errata (94; 0.21%), letters (155; 0.34%), notes (149; 0.33%), retracted publications (17; 0.04%), and short surveys (61; 0.13%).

**Figure 3:**
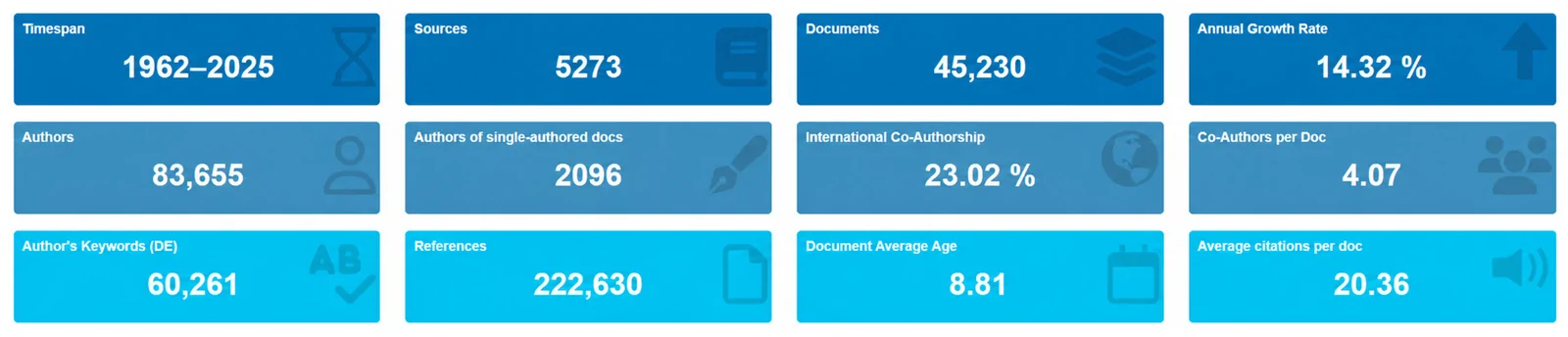
The overall status of research in the field of eye tracking.

The use of 77,596 Keywords Plus and 60,261 author keywords across the publications suggests considerable conceptual diversity in eye-tracking research and its association with different research themes. The completeness of the citation metadata in the dataset was also assessed. Cited References (CR) information was available for 43,314 of the 45,230 records (95.76%), whereas this field was missing for 1916 records (4.24%). The “References” value reported as 222,630 in the Bibliometrix output was not treated as the number of unique references (Figure 3).

According to Scopus data, the concentration of eye-tracking research in Computer Science (19.8%) suggests a strong association with computer science- and technology-oriented research [46]. This distribution can be considered in relation to its connections with fields such as artificial intelligence, computer vision, human–computer interaction, and data analytics. The high representation of Medicine (12.2%), Neuroscience (10.7%), and Psychology (9.4%) indicates that eye-tracking methods have become not merely a technical tool but one of the fundamental research methods for studying cognitive processes, visual attention, and human behavior. Similarly, the concentration in the fields of Engineering (10.5%) and Social Sciences (9.0%) demonstrates that eye-tracking technologies have been widely adopted both in experimental research and in solving applied problems. Mathematics (4.7%) and Arts and Humanities (4.7%) also contribute to the distribution, while Biochemistry, Genetics and Molecular Biology (3.1%) and Physics and Astronomy (2.8%) have a more limited representation. When the distribution across fields is evaluated as a whole, it becomes clear that eye-tracking research functions as a methodological bridge between different fields of knowledge rather than being a research area confined to the boundaries of a single discipline. In particular, the high representation of both technical sciences and behavioral and health sciences suggests that eye-tracking technologies have evolved into an interdisciplinary research infrastructure that enables the analysis of human behavior through objective and measurable data. The fields grouped under “Other” account for 13.0% of the publications, further indicating the diversity of subject areas represented in the dataset. These percentages are calculated based on the total number of publications in the dataset (*n* = 45,230). Because Scopus may assign a single publication to more than one subject area, the percentages are not mutually exclusive and therefore are not expected to sum to 100%.

**Figure 4:**
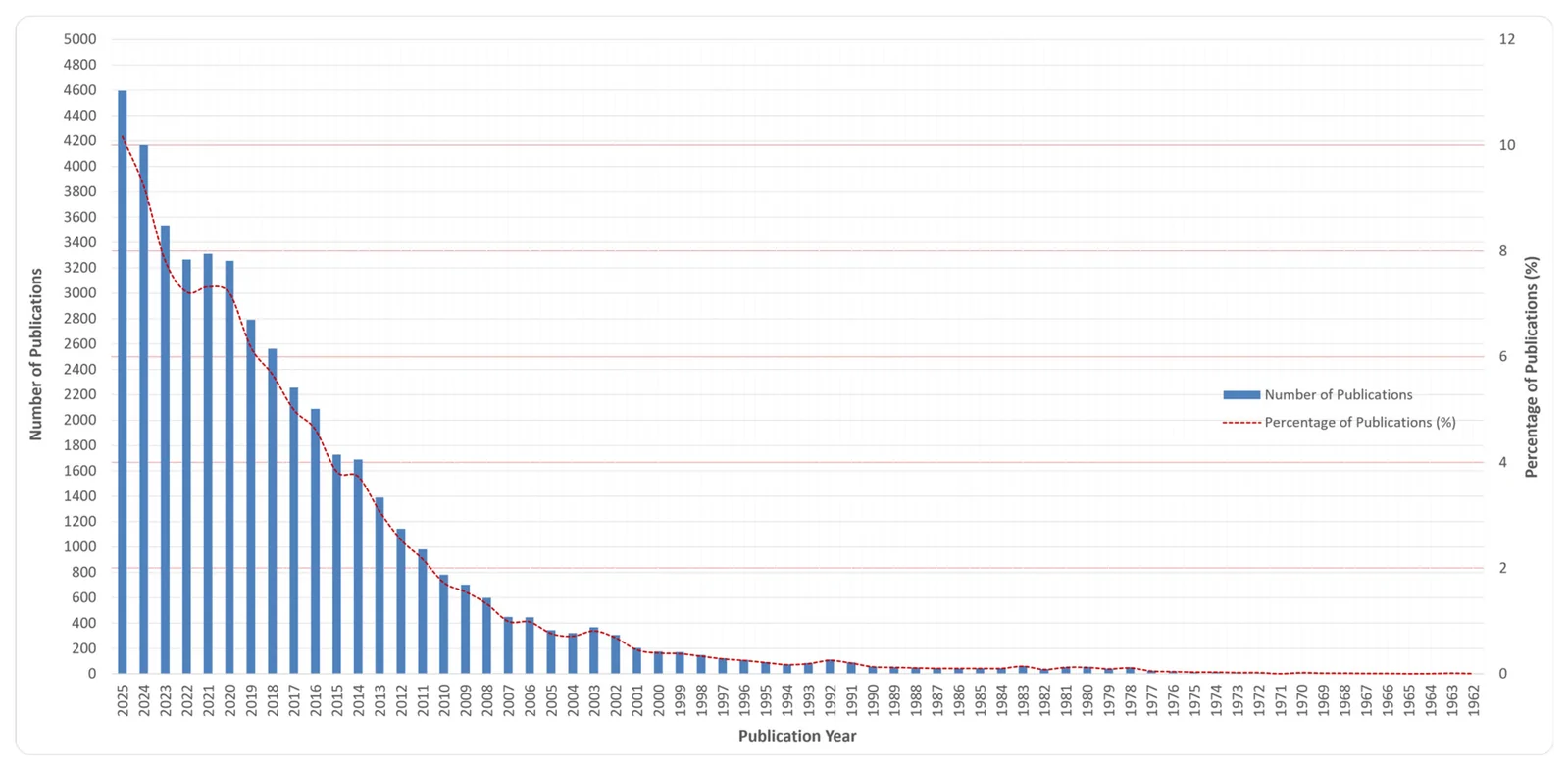
Annual publication output in eye-tracking research (Bibliometrix/Biblioshiny; 1962–2025; number of publications).

Findings from the Scopus database indicate that scientific output in eye-tracking research has shown a long-term growth trend. The first publication in this field dates back to 1962, and publication numbers remained relatively low in the following years. However, a marked upward trend in eye-tracking research has been observed since the 2000s. The annual number of publications reached 176 in 2000, 780 in 2010, and 1729 in 2015. This upward trend continued in subsequent years; in particular, after 2020, annual publication output exceeded 3000, demonstrating the field’s continued rapid development. Indeed, the annual number of publications was 3311 in 2021, 3265 in 2022, 3534 in 2023, 4169 in 2024, and 4595 in 2025 (Figure 4).

When the findings are considered, 23,100 publications were produced in the eye-tracking field over the 58-year period from 1962 to 2019, whereas 22,130 publications, corresponding to approximately 49% of the total publications, were published during the 2020–2025 period alone. This distribution suggests that eye-tracking research has experienced notable growth in recent years, with scientific output increasingly concentrated within shorter periods (Figure 4).

### 3.1. Analysis of Scientific Production, Temporal Evolution, and International Collaboration by Country

**Figure 5:**
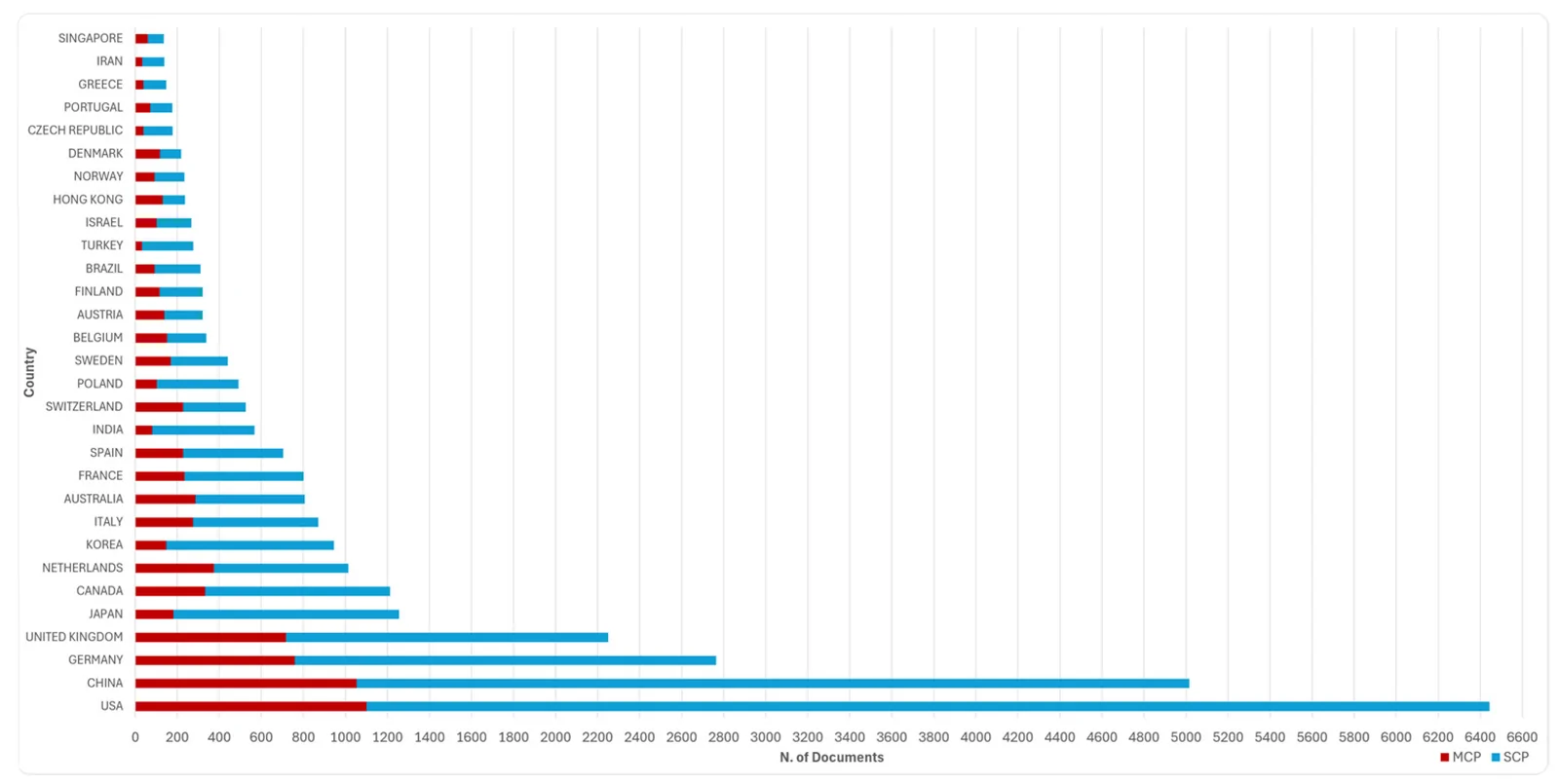
Country distribution and publication output of corresponding authors (Bibliometrix/Biblioshiny; SCP: single-country publications; MCP: multiple-country publications).

By examining the countries of the corresponding authors and the number of articles, this study identified the countries where research in the field of eye tracking is concentrated and the levels of international collaboration among countries. When examining the publications represented in the graph as SCP (single-country publications) and MCP (multi-country publications), it is evident that the United States, with 6444 studies (SCP: 5343, MCP: 1101), and China, with 5016 studies (SCP: 3963, MCP: 1053), rank among the top (Figure 5; Table 2). The fact that both countries have high values in both single-country and multi-country publications indicates not only their strong research production capacity but also their active roles in international research networks. In particular, the high MCP values indicate that eye-tracking research is increasingly being conducted through international collaborations. This suggests that international collaboration is a notable feature of the analyzed eye-tracking literature and may reflect the field’s connection to global research networks.

**Figure 6:**
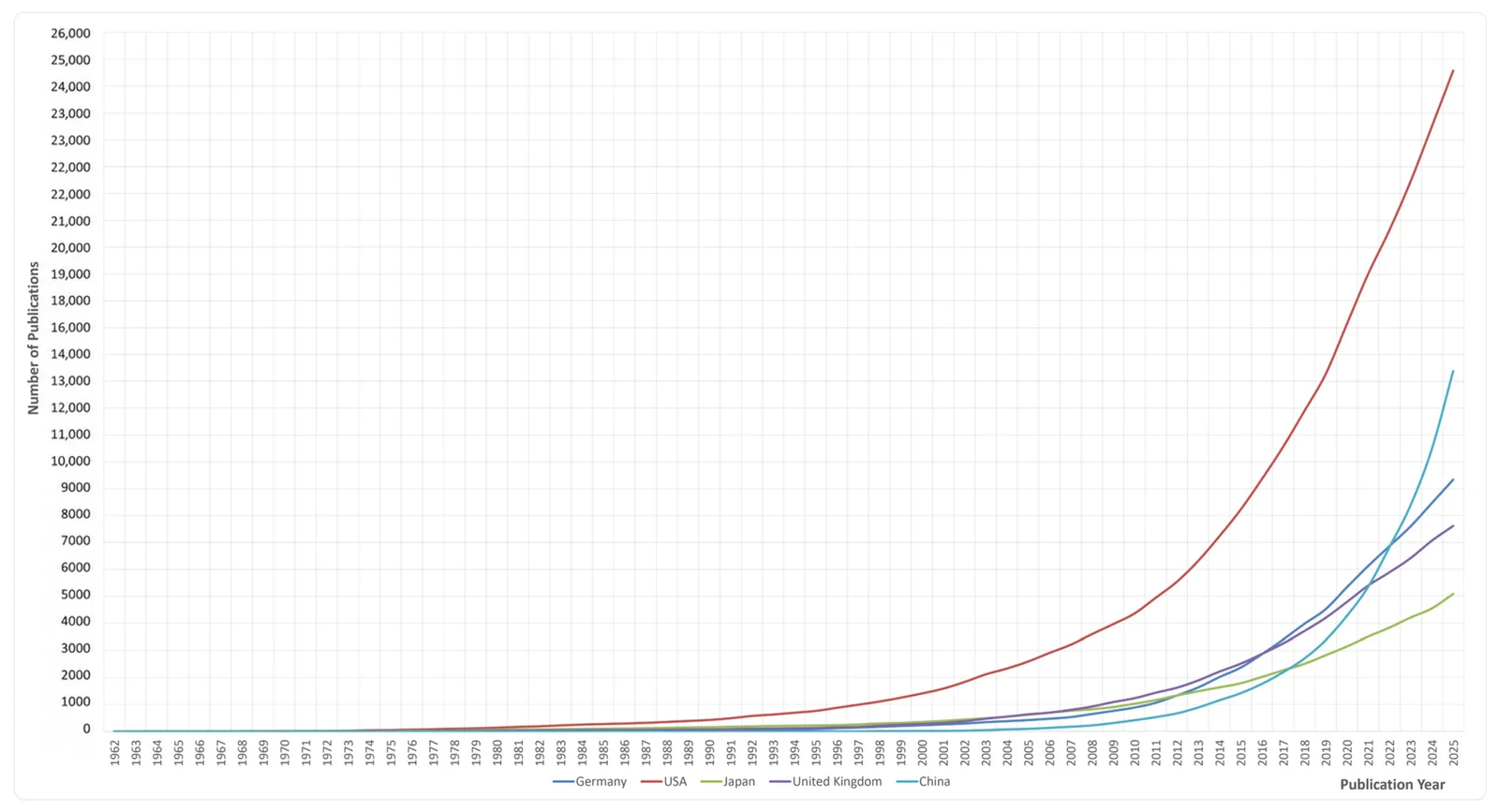
Countries’ publication output over time (Bibliometrix/Biblioshiny; 1962–2025; number of publications).

When the scientific production of countries over time is examined, the United States appears to have been the most productive country in the field from the early years of the study period and to have maintained this position throughout the period (Figure 6). In contrast, China’s scientific output remained relatively limited for many years but increased notably, particularly from the 2010s onward, and surpassed Germany after 2022 to rank second. Germany, the United Kingdom, and Japan showed more consistent and gradual growth throughout the study period, without a sharp increase comparable to that observed in China. These findings suggest that the growth of scientific production in eye-tracking research involved not only a quantitative increase over time but also changes in the geographical distribution of research activity and the relative levels of scientific output across countries. The overall distribution of country affiliations further shows that the United States, Germany, the United Kingdom, and China were among the leading countries (Figure 6 and Table 3). Table 3 shows that the United States ranked first in country-level scientific production, with 24,575 country affiliations, followed by Germany (13,373) and the United Kingdom (9336). The United States also recorded the highest total citations (222,315) and average article citations (34.5) among the countries listed. The Netherlands (32.9), Denmark (30.7), and the United Kingdom (30.0) followed in terms of average article citations.

**Figure 7:**
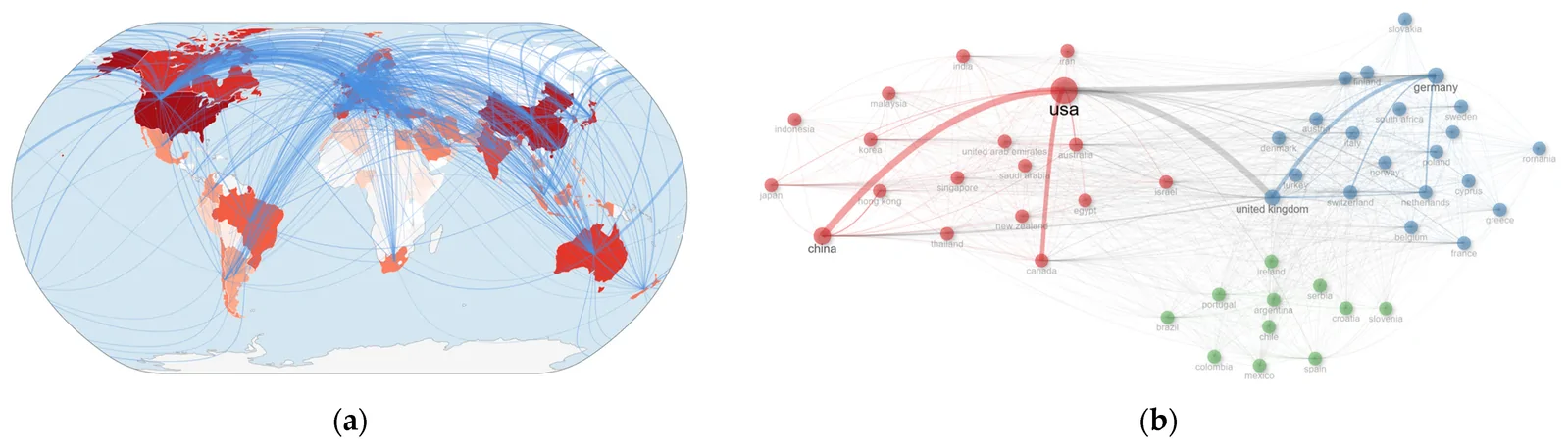
International collaboration in eye-tracking research: (**a**) Country collaboration map (Bibliometrix/Biblioshiny; minimum two co-publications). (**b**) Country collaboration network (Bibliometrix/Biblioshiny; 50 nodes; minimum one edge).

**Figure 8:**
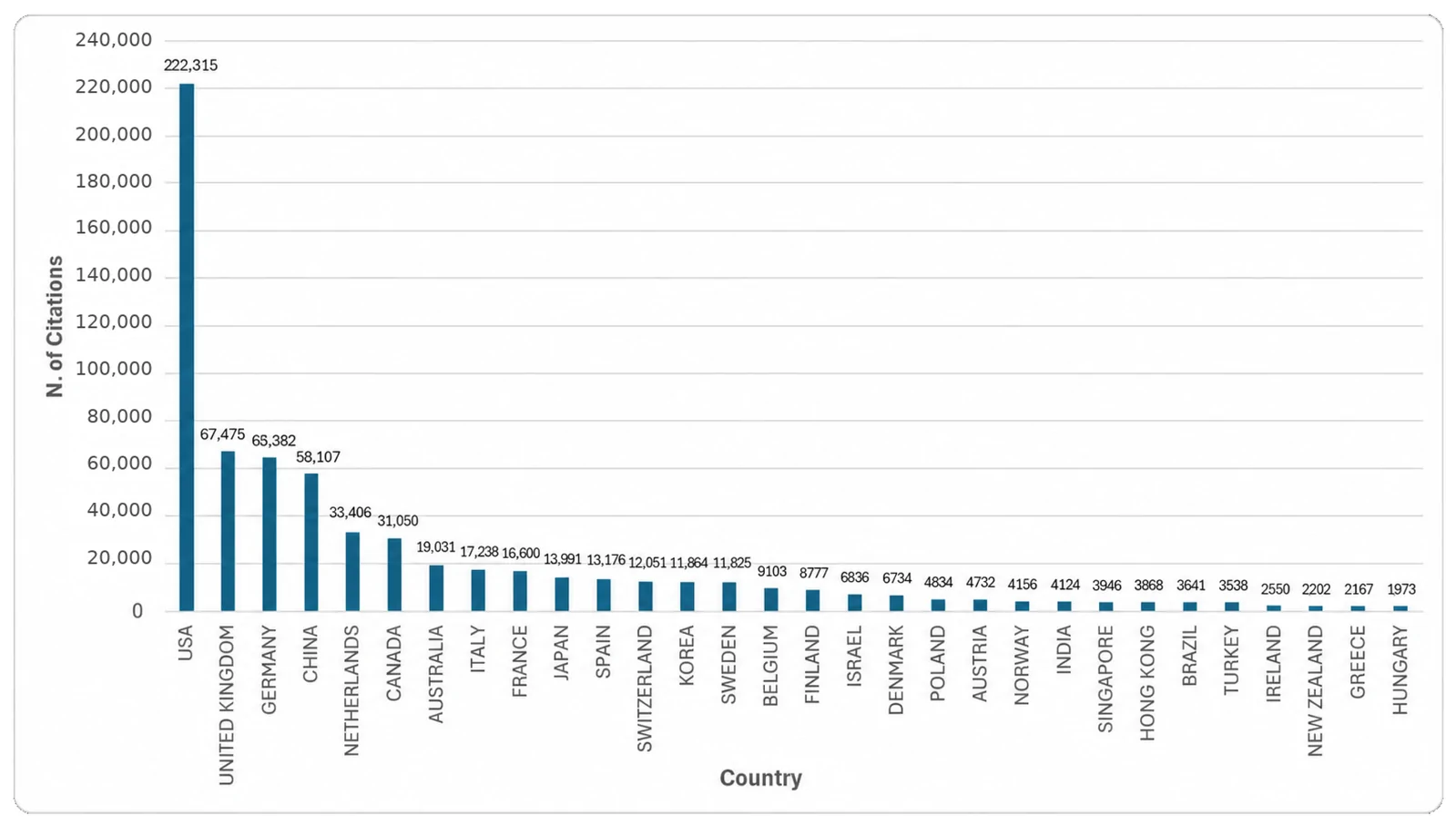
Top 30 countries by total citation count (Bibliometrix/Biblioshiny; total citations).

Among the collaboration networks of countries engaged in scientific research in the field of eye tracking, the most intensive collaborations occurred between the United States and China (*n* = 604), the United States and the United Kingdom (*n* = 585), the United States and Germany (*n* = 577), and the United States and Canada (*n* = 480). In particular, the United States occupies the central position in the global collaboration network, maintaining a high frequency of joint publications with numerous countries. Germany also stands out in Europe-centered collaborations through its strong collaborative links with the United Kingdom (*n* = 452) and the Netherlands (*n* = 302). Furthermore, China and Australia have made significant contributions to the expansion of the collaboration network through partnerships established with countries across different regions (Figure 7 and Figure 8). The resulting network structure suggests that international collaboration plays a decisive role in eye-tracking research and that the field is shaped by interconnected research communities on a global scale.

### 3.2. Institutional Scientific Production, Collaboration, and Temporal Evolution

**Figure 9:**
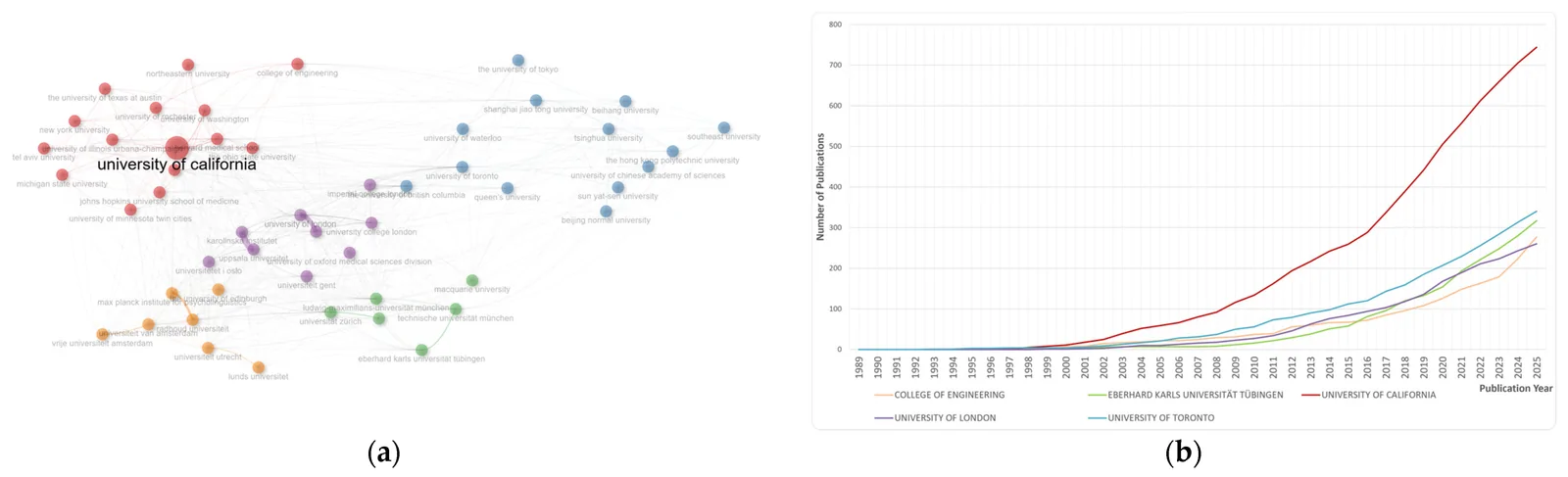
Institutional publication analysis: (**a**) Collaboration network among the most productive institutions (Bibliometrix/Biblioshiny; 50 nodes; minimum one edge). (**b**) Temporal evolution of institutional publication output (Bibliometrix/Biblioshiny; number of publications over time).

**Figure 10:**
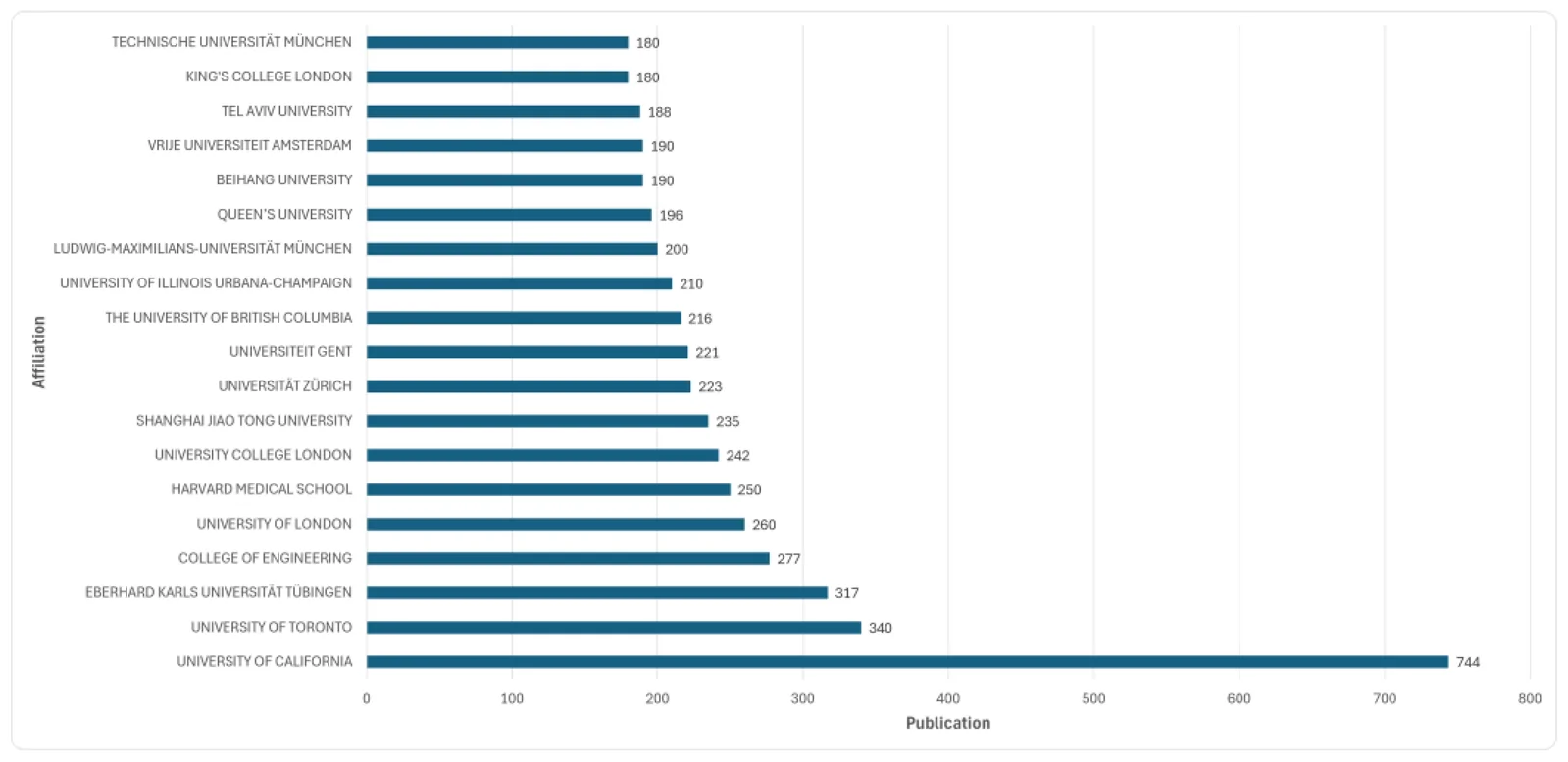
Top 20 most productive institutions (Bibliometrix/Biblioshiny; Most Relevant Affiliations).

The inter-institutional collaboration network (Figure 9a), the publication output of the top five countries over time (Figure 9b), and the top 20 institutions producing the most publications in the field of eye tracking (Figure 10) were examined.The findings show that, based on Scopus affiliation records, the University of California (*n* = 744) had the highest number of publications and maintained its leading position throughout the study period. However, this result should be interpreted with caution because Scopus may represent multiple campuses and affiliated units of the University of California system under a single institutional record. Accordingly, the institutional ranking reflects the structure of Scopus affiliation records and should not be interpreted as a direct comparison of individual campuses. In the inter-institutional collaboration network, the University of California plays a central role, with U.S.-based universities in particular forming strong connections; meanwhile, leading institutions in Europe and Asia cluster within their own regions but are integrated into the global network through these central institutions. These findings suggest that the institutions prominent in eye-tracking research also occupy active positions within international scientific collaboration networks. However, institutional productivity and collaboration findings should be interpreted with consideration of differences in institutional naming and hierarchical structures within Scopus affiliation data.

### 3.3. Author Productivity and Temporal Evolution

**Figure 11:**
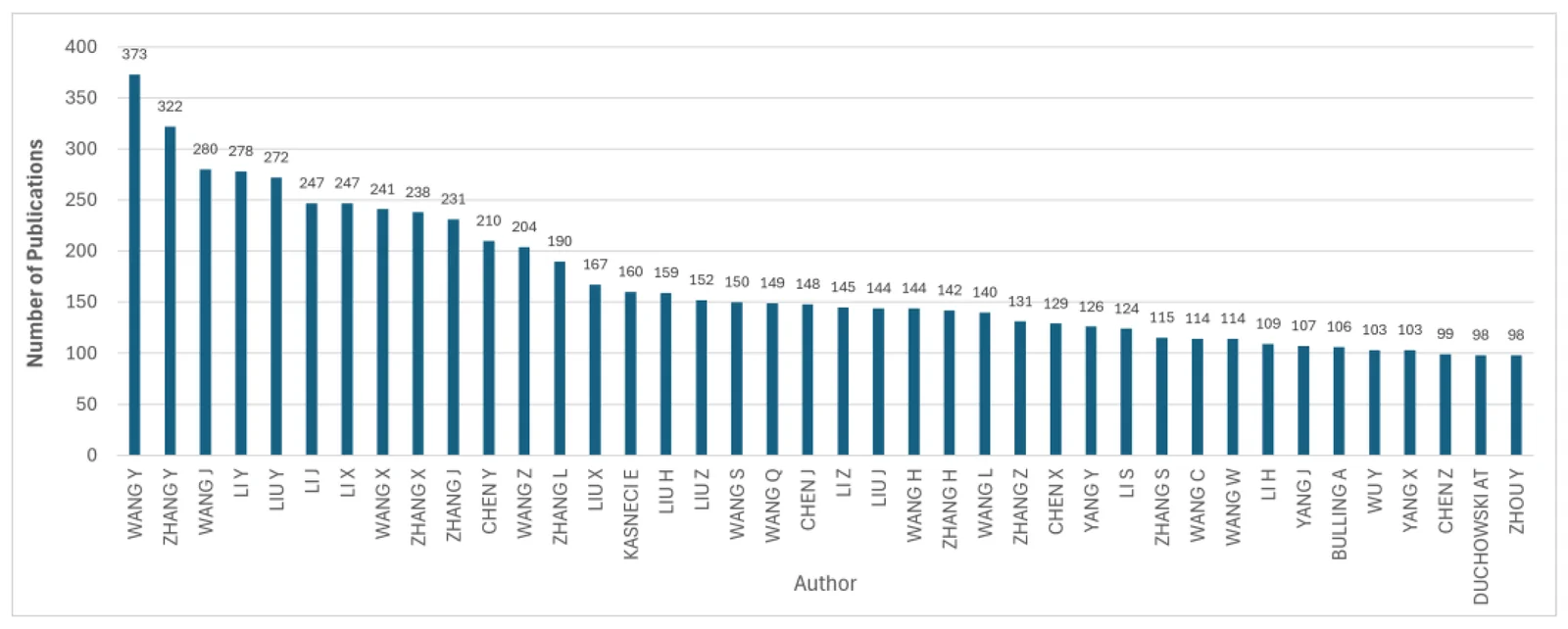
Top 40 authors by publication output (Bibliometrix/Biblioshiny; Most Relevant Authors).

**Figure 12:**
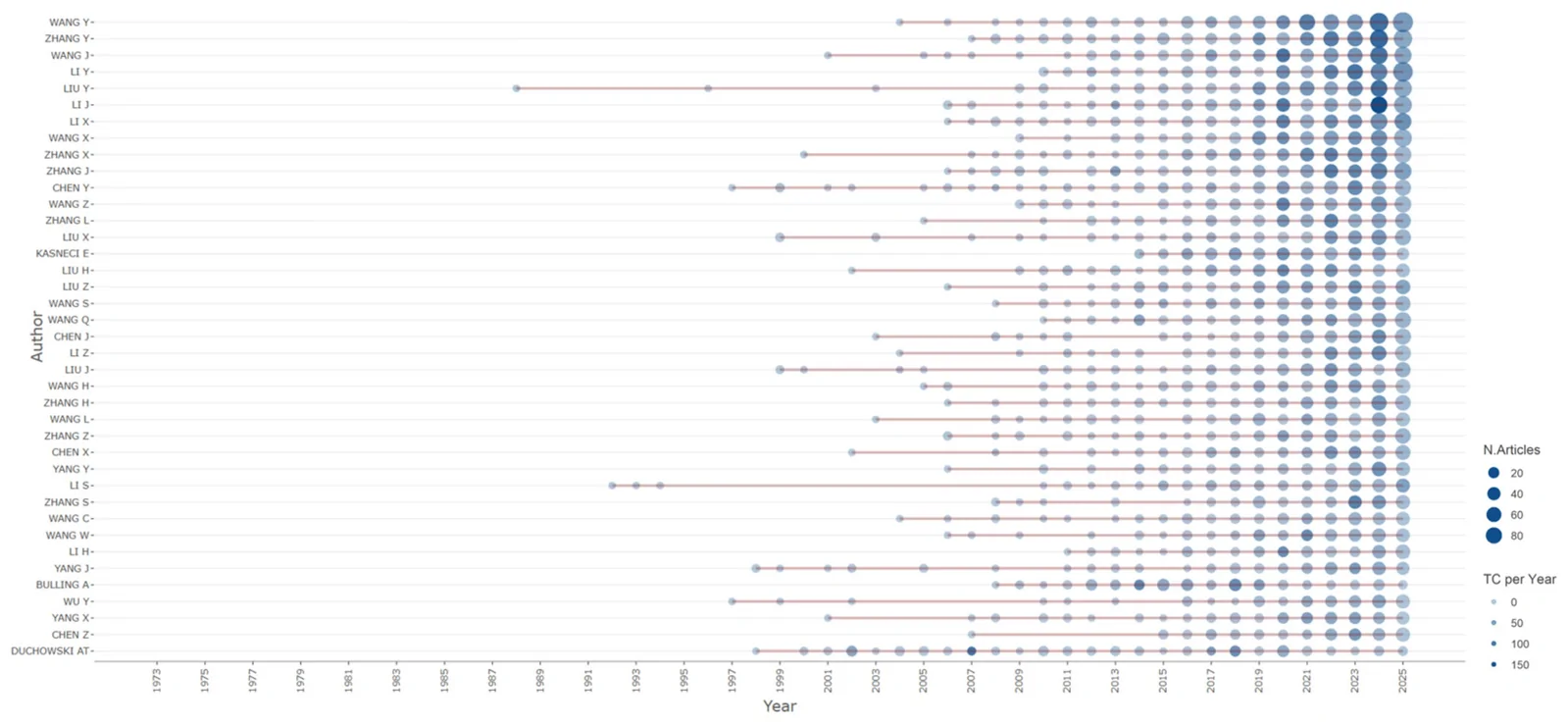
Authors’ production over time (Bibliometrix/Biblioshiny).

The top 40 groups of authors with the highest number of publications in the field of eye tracking are presented in Figure 11, while authors’ publication output over time is shown in Figure 12. Due to the default matching system of the Bibliometrix/Biblioshiny software, authors have been grouped by last name and initials. Therefore, the results should be interpreted in terms of author labels rather than individual researchers. Accordingly, the rankings shown in Figure 11 and Figure 12 refer to author labels rather than uniquely identified individual researchers.

The findings indicate that the author labels Wang Y (*n* = 373), Zhang Y (*n* = 322), Wang J (*n* = 280), Li Y (*n* = 278), and Liu Y (*n* = 272) have the highest number of publications. When examining the timeline of author productivity, some author labels exhibit publication histories spanning many years. However, different researchers with the same surname and initials may be grouped under the same author label; therefore, these results should not be interpreted as individual author performance. This issue may be particularly pronounced for surnames such as Wang and Zhang, which are common in Chinese naming conventions, and may result in different researchers being grouped under the same author label. Therefore, potential limitations arising from name disambiguation should be considered when interpreting the findings for the most productive author labels.

The increase in dot size and color intensity toward the most recent years in Figure 12 indicates that publication and citation output in eye-tracking research has become increasingly concentrated. This suggests that the field has grown significantly in recent years and that its scientific visibility has increased.

During the examination of the author data, 731 records were found to have the author name field marked as NA. These records were excluded from the ranking of authors by publication productivity. The missing records were considered one of the limitations of the author metadata in the dataset.

### 3.4. Source Productivity and Temporal Evolution

**Figure 13:**
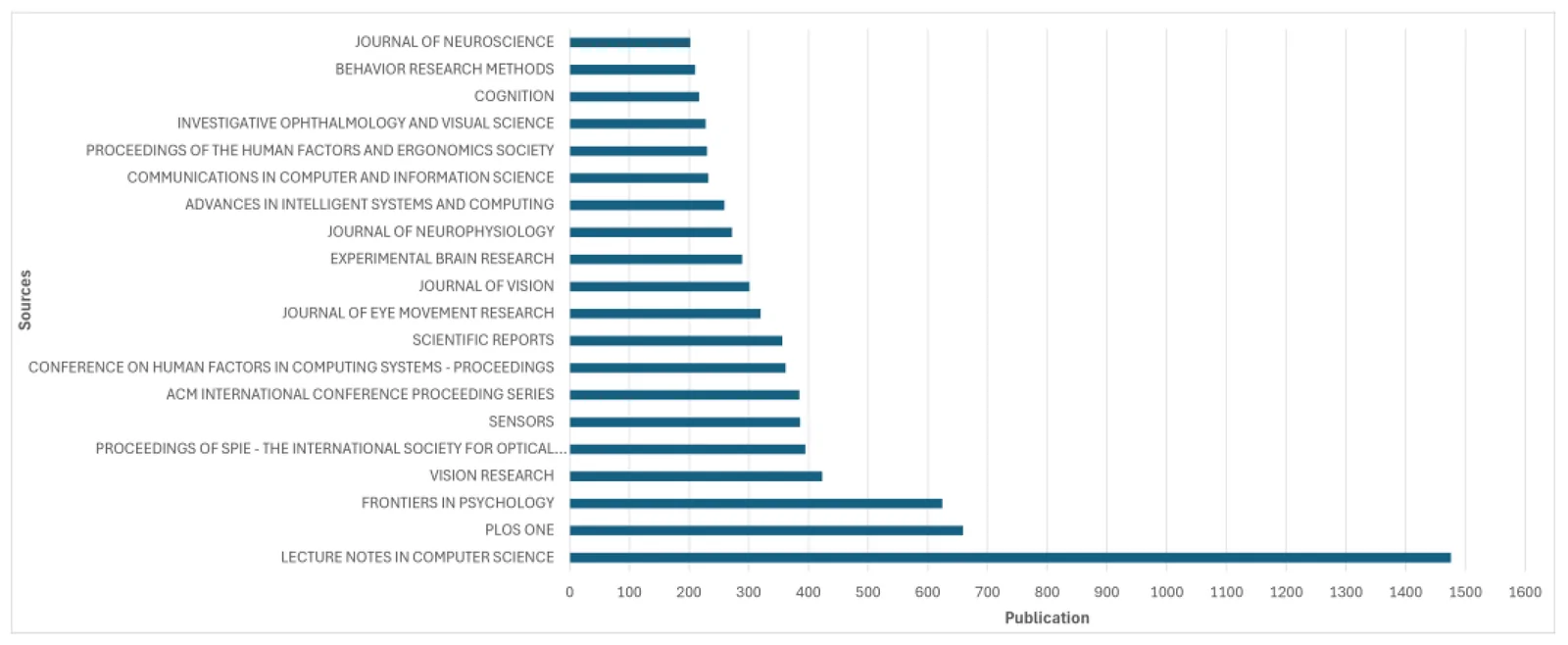
Top 20 sources by publication output (Bibliometrix/Biblioshiny; Most Relevant Sources).

**Figure 14:**
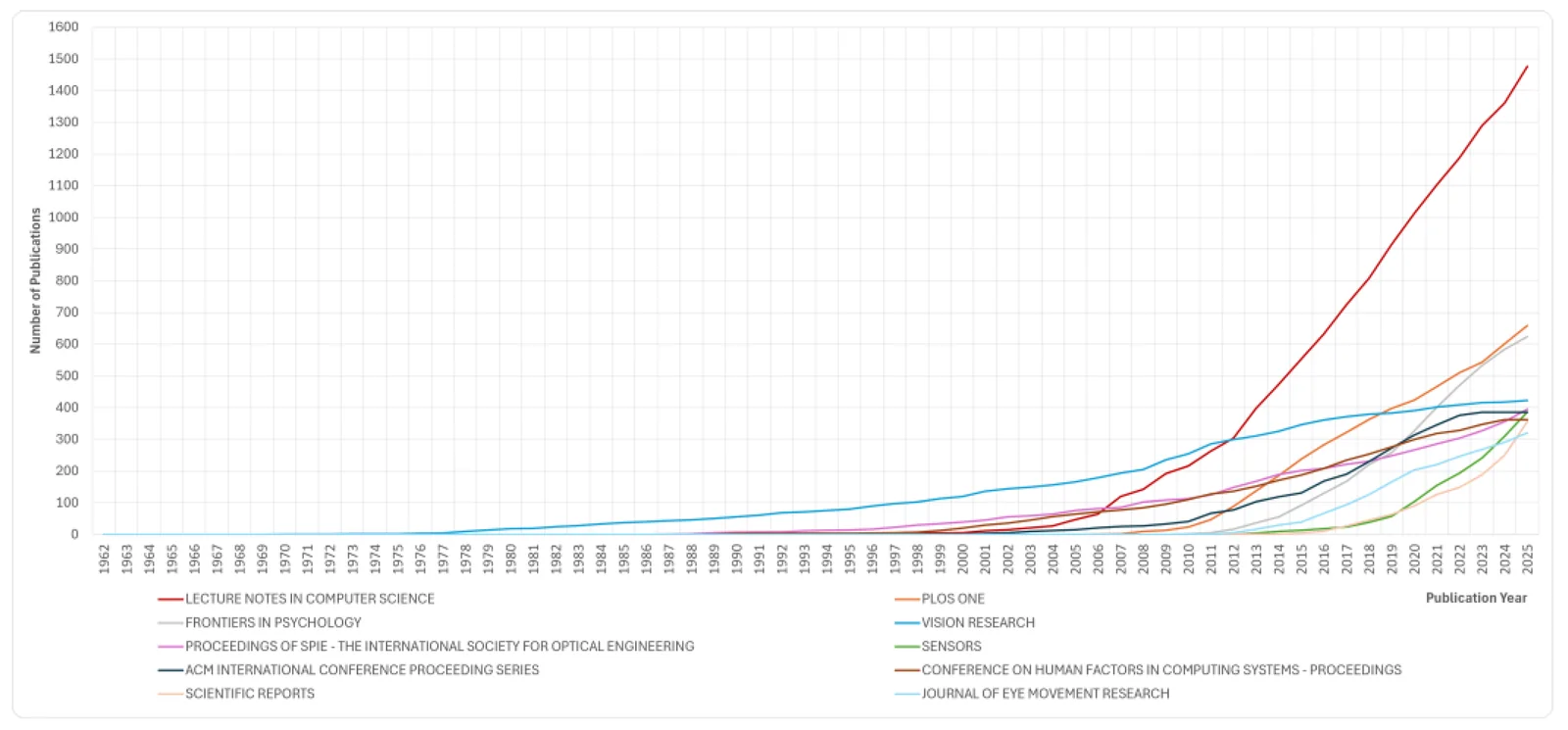
Scientific production of the top five sources over time.

The top 20 sources with the highest number of publications in the field of eye tracking (Figure 13) and the publication output of the top 5 sources over time (Figure 14) are shown. Lecture Notes in Computer Science (LNCS) (*n* = 1476) had the highest publication output among the sources shown. It is followed by interdisciplinary, high-impact journals such as PLOS ONE (*n* = 659) and Frontiers in Psychology (*n* = 624). It is also noteworthy that vision- and neuroscience-focused journals such as “Vision Research” (*n* = 423), “Journal of Eye Movement Research” (*n* = 320), “Journal of Vision” (*n* = 301), and “Experimental Brain Research” (*n* = 289) account for a significant share. The presence of ACM and SPIE conference proceedings as important publication channels also suggests that eye-tracking research is particularly represented in publication venues associated with computer science, human–computer interaction, and engineering. This distribution suggests that eye-tracking research is represented across both journal- and conference-based publication channels and spans multiple disciplinary areas.

### 3.5. Citation and Co-Citation Analysis of Publications

**Figure 15:**
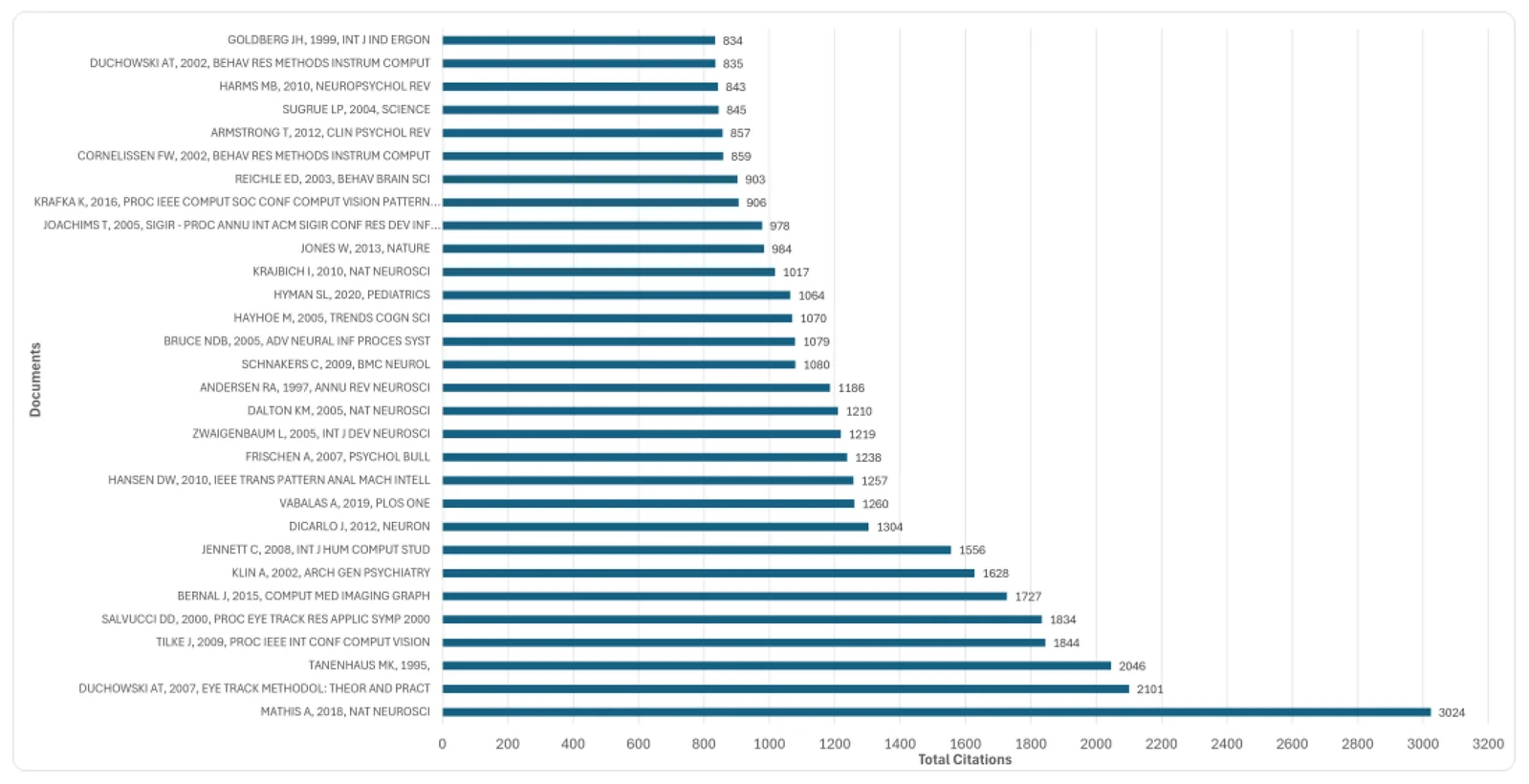
Most Global Cited Documents (Bibliometrix/Biblioshiny; top 30 documents by total citation count).

The publications with the highest number of citations globally in the field of eye tracking were examined (Figure 15). The global citation count refers to the total number of citations a publication has received from all publications indexed in databases such as Scopus and Web of Science [51]. The findings show that the studies with the highest global citation counts among the publications in the dataset are those by A. Mathis (*n* = 3024), A.T. Duchowski (*n* = 2101), and M.K. Tanenhaus (*n* = 2046). However, it was determined that although A. Mathis’s study includes the concept of eye tracking among the keywords indexed by Scopus, it does not focus directly on eye-tracking methods but is related to the field of behavior and motion tracking. This situation demonstrates that keyword-based queries used in broad bibliometric searches may also include some studies that are only indirectly related to the research topic. In contrast, A.T. Duchowski’s book “Eye Tracking Methodology: Theory and Practice” and M.K. Tanenhaus’s studies on eye-tracking applications are considered fundamental references in the field because they focus directly on eye-tracking methodology and applications. This finding suggests that studies related to methodological and theoretical foundations also constitute an important part of the highly cited publications.

**Figure 16:**
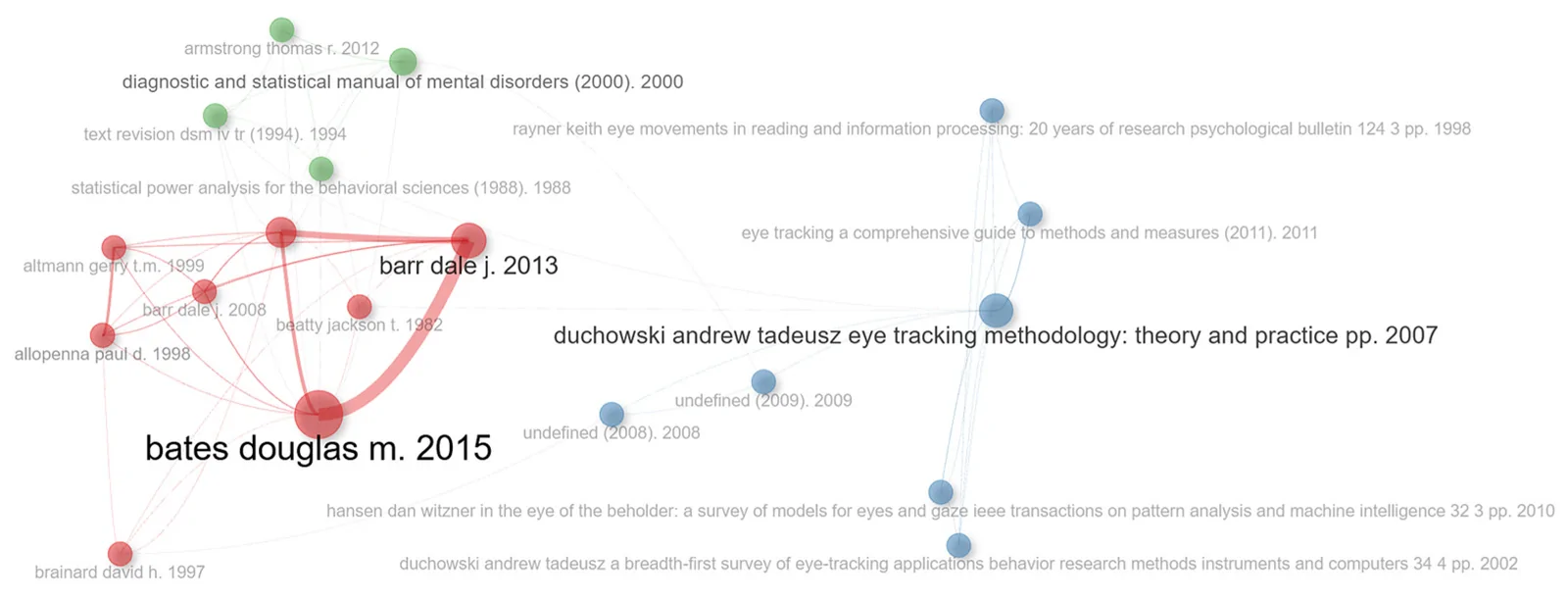
A co-citation network of eye-tracking publications (Bibliometrix/Biblioshiny; 20 sources with the highest number of links; minimum 5 links).

To examine the intellectual structure of the publications, a co-citation analysis was conducted (Figure 16). The analysis examined sources that were cited together in the reference lists of publications in the dataset. For network visualization, the 20 sources with the highest number of links were included, and a minimum link threshold of 5 was applied. The resulting network shows a distinct cluster of studies related to eye-tracking methodology and eye movements. Within this cluster, Duchowski’s Eye Tracking Methodology: Theory and Practice (2007) [52] stands out, reflecting the position of eye-tracking methodology within the intellectual foundation of the field. In addition, the presence of sources related to psychology, attention, clinical assessment, and statistical methods in other clusters suggests that eye-tracking research has developed within an intellectual structure connected to diverse disciplinary and methodological sources. Of the 20 sources displayed in the network, 2 (10%) had undefined bibliographic information. As an additional check, a broader network using 500 nodes and a minimum link threshold of 1 identified 25 sources with undefined bibliographic information. These undefined records were taken into account when interpreting the network, and the cluster-level findings were interpreted primarily in terms of broader thematic relationships among the identifiable sources.

### 3.6. Keyword, Thematic, and Three-Field Plot Analysis

**Figure 17:**
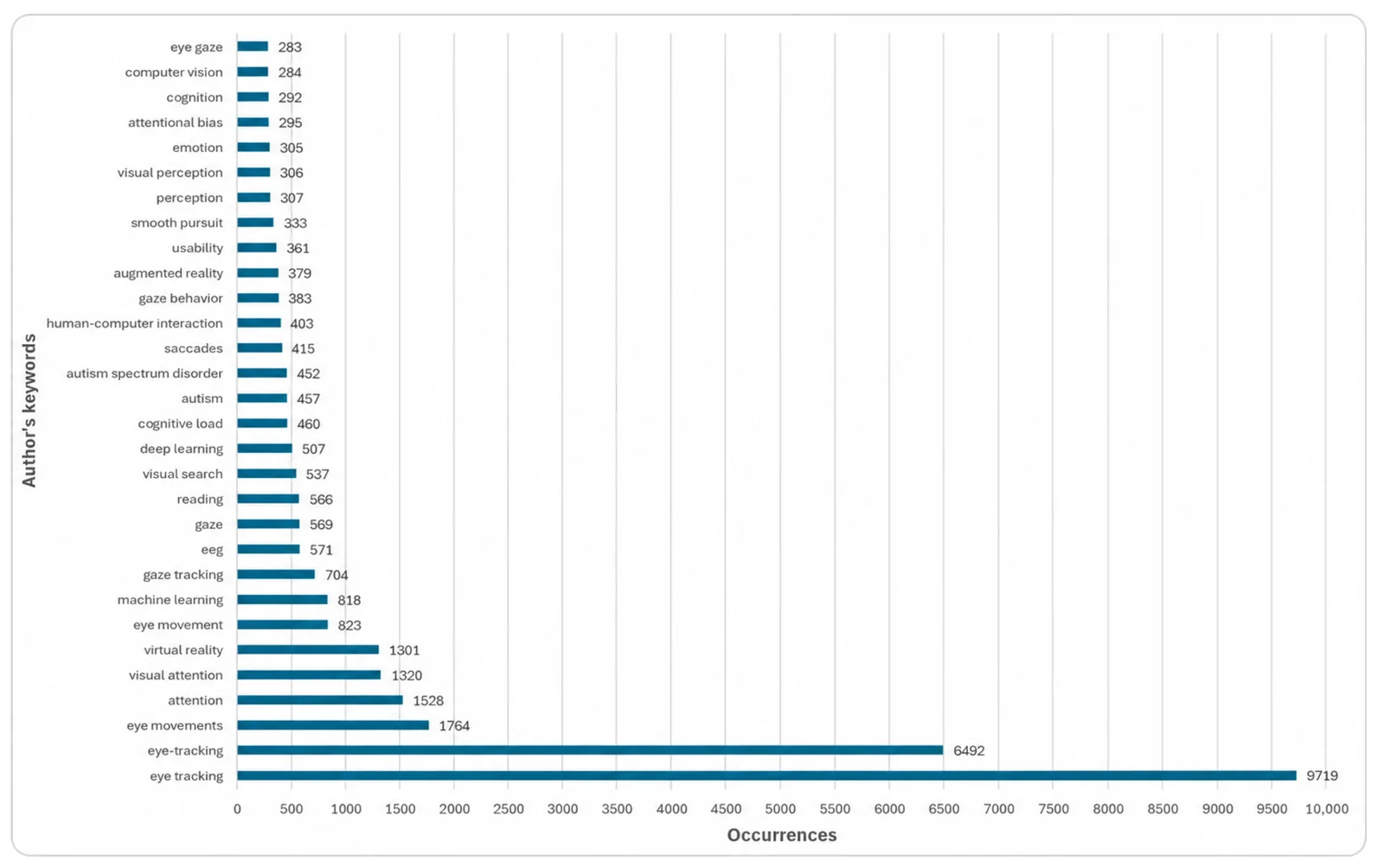
Top 30 most frequently used keywords (Bibliometrix/Biblioshiny; Most Frequent Words; Author’s Keywords).

The most frequently used keywords in the field of eye tracking were examined (Figure 17). It was found that, after the terms “eye tracking” and “eye movements,” the concepts of “attention” and “visual attention” stood out as the most frequently used keywords. The thematic classification of these keywords and their associated countries is presented in Table 4. The classification covers several thematic areas, including eye-tracking fundamentals, visual attention and cognitive processes, artificial intelligence and computer vision, human–computer interaction, and virtual and extended reality. This suggests that eye tracking may play an important role in research examining cognitive processes. Terms such as virtual reality, human-computer interaction, and gaze behavior indicate that the technological and applied aspects of the field have gained prominence; meanwhile, the inclusion of artificial intelligence-based approaches—such as machine learning and deep learning—among the keywords suggests that eye-tracking data is being integrated with advanced analytical methods. Keywords related to application areas such as autism, cognitive load, and reading, meanwhile, indicate that eye tracking is widely used in clinical and behavioral research. Overall, the distribution of keywords demonstrates that eye tracking studies are multidisciplinary, methodologically advanced, and characterized by a high degree of application diversity.

**Figure 18:**
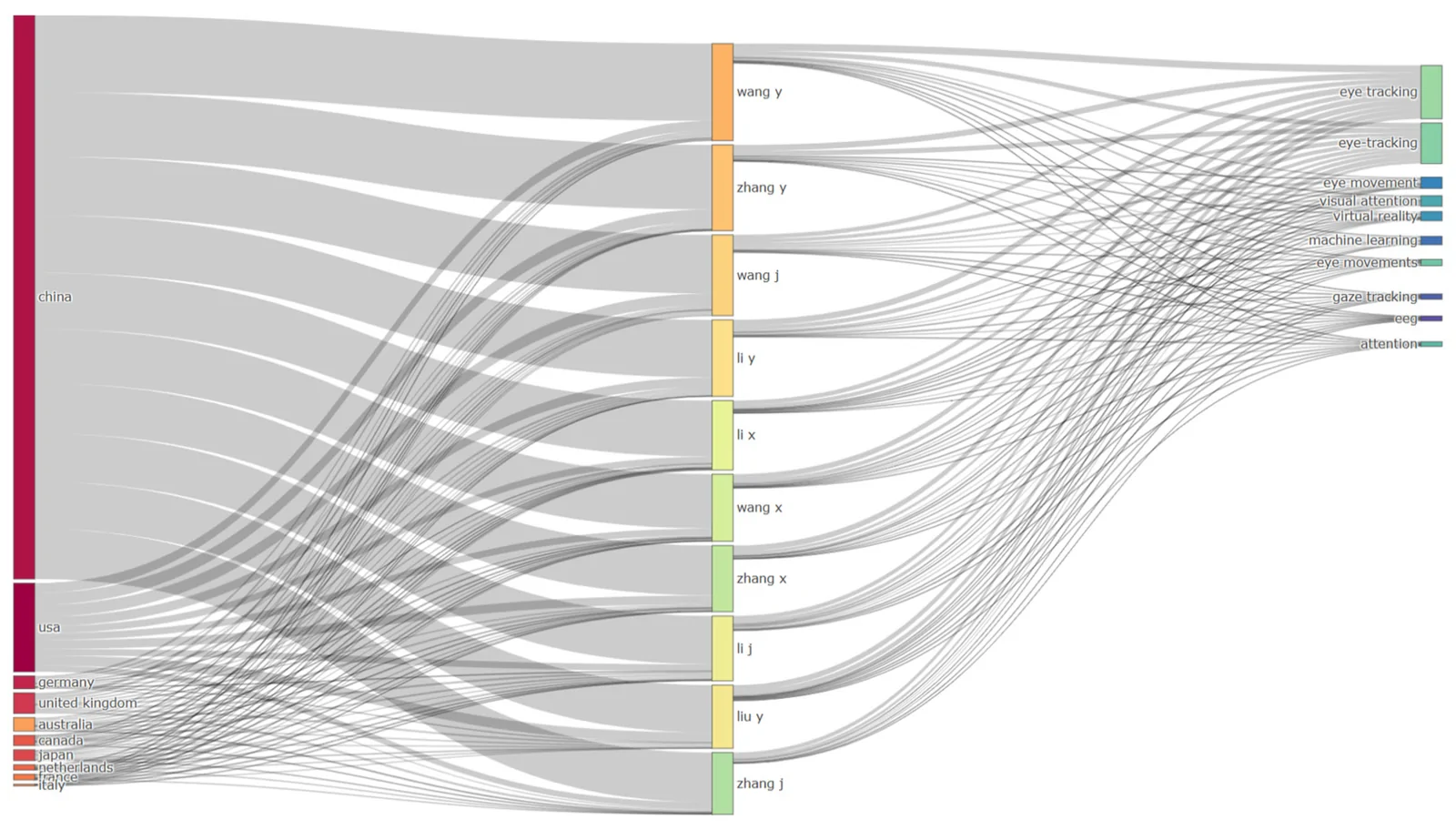
Three-field plot of countries, authors, and keywords in eye-tracking research (Bibliometrix/Biblioshiny; top 10 countries, top 10 authors, and top 10 keywords).

Biblioshiny enables analyses of three-field relationships (e.g., countries, authors, institutions, sources, titles, abstracts, and keywords). In this study, the relationships among the top 10 countries, authors, and keywords in the eye-tracking literature were analyzed (Figure 18). The findings indicate that publication output is primarily concentrated in China, followed by the United States, Germany, the United Kingdom, Japan, and Australia. The author labels Wang Y., Zhang Y., Wang J., Li Y., Li X., and Wang X. appear prominently in the three-field plot and are associated with multiple thematic areas. It should be noted that these author labels are based on surname–initial combinations and may therefore represent more than one individual author. Keyword analysis reveals that the literature focuses not only on fundamental concepts such as eye tracking, eye movements, and visual attention but also on technological and interdisciplinary topics, including EEG (electroencephalography), virtual reality, and machine learning. These findings suggest relationships among countries, authors, and research topics, highlighting the interdisciplinary nature of eye-tracking research and its expansion toward emerging technological approaches while maintaining its core research themes.

### 3.7. Trend Topic Analysis

**Figure 19:**
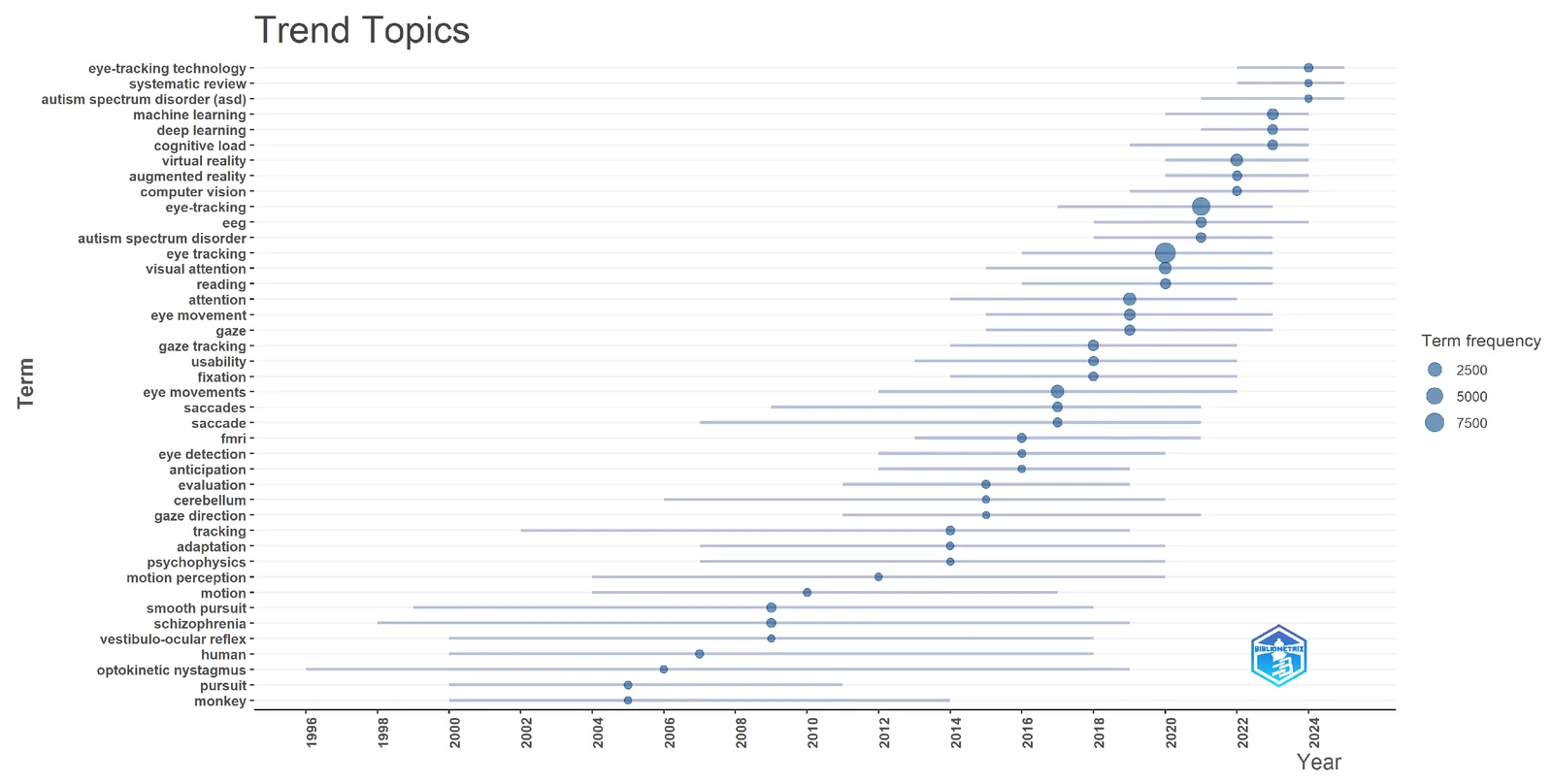
Trend topics in eye-tracking research (Bibliometrix/Biblioshiny; Author’s Keywords; minimum word frequency = 50; maximum three terms per year).

To identify the research topics and thematic trends that have gained prominence over time in eye-tracking research, a Trend Topics analysis was conducted using Biblioshiny (Figure 19). The Author’s Keywords field was used, and the analysis period was set to 1962–2025. The minimum word frequency was set to 50, and a maximum of three terms was displayed for each year. No term removal or thesaurus was applied. Based on these settings, the Trend Topics results are presented in Figure 19.

The Trend Topics analysis shows that the prominent research topics in eye-tracking research have changed over time. In the early period, terms such as pursuit, optokinetic nystagmus, vestibulo-ocular reflex, smooth pursuit, and monkey were prominent, whereas in later years, terms such as motion perception, tracking, adaptation, gaze direction, eye detection, fixation, saccades, and eye movements became more prominent. This pattern suggests that research topics in the early period were primarily centered on eye movements and oculomotor processes.

After 2018, terms such as gaze tracking, usability, fixation, gaze, eye movement, attention, and visual attention became more prominent. During this period, the research focus appears to have expanded beyond eye movements to include visual attention, gaze behavior, and usability.

After 2020, terms such as eye tracking, EEG, virtual reality, augmented reality, computer vision, cognitive load, deep learning, and machine learning became more prominent in the graph. The emergence of these terms in recent years may indicate that technological and computational approaches have become increasingly visible in eye-tracking research.

In the most recent part of the graph, eye-tracking technology, systematic review, and autism spectrum disorder (ASD) are among the prominent terms. This finding suggests that the recent research agenda includes not only eye-tracking technologies but also systematic reviews and specific application areas.

### 3.8. Thematic Map Analysis

**Figure 20:**
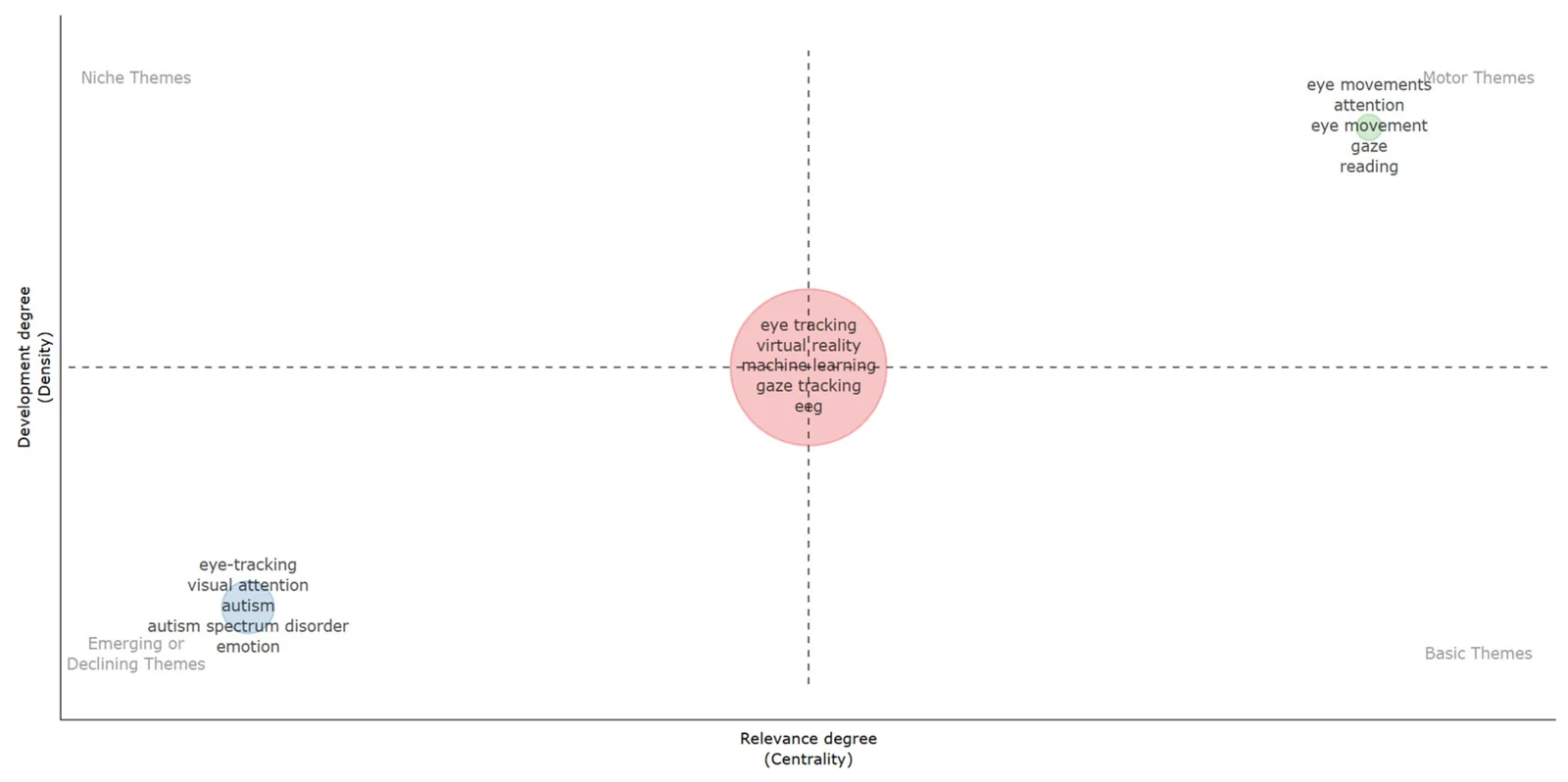
Thematic map of eye-tracking research (Bibliometrix/Biblioshiny; Author’s Keywords; minimum frequency = 5; 250 keywords).

The thematic map shows that different thematic clusters in eye-tracking research are positioned differently in terms of centrality and development (Figure 20). The Motor Themes quadrant includes the terms “eye movements,” “attention,” “eye movement,” “gaze,” and “reading.” This cluster exhibits high centrality and density, indicating stronger connections with other themes in the field and a relatively more developed thematic structure. The terms “eye tracking,” “virtual reality,” “machine learning,” “gaze tracking,” and “EEG,” which are positioned around the intersection of the centrality and density axes, represent a thematic cluster located at moderate levels on both dimensions. The terms “Eye-tracking,” “visual attention,” “autism,” “autism spectrum disorder,” and “emotion” are located in the Emerging or Declining Themes quadrant. This position alone does not allow the developmental level or current relevance of these themes within the field to be determined; however, it shows that these themes occupy a different thematic position from the Motor Themes. The absence of clearly defined Niche Themes or Basic Themes in the map also suggests that the keywords in the dataset are concentrated within certain thematic clusters, while some themes are not clearly differentiated from the others.

### 3.9. Sensitivity Analysis of Off-Topic Records

To assess whether the presence of potentially off-topic records affected the principal bibliometric findings, a rule-based sensitivity analysis was conducted on the full dataset using R. The rule-based procedure was initially developed and calibrated using a manually validated random sample of 300 records. Of these records, 257 (85.67%) were classified as within scope, whereas 43 (14.33%) were classified as off-topic. This initial assessment provided an estimate of the potential contamination of the dataset and indicated that off-topic records constituted a non-negligible proportion of the full corpus.

The rule-based screening relied on a predefined set of eye-tracking-related terms, including terms referring to eye tracking, gaze tracking, eye movements, visual fixation, fixation duration, saccades, and pupillometry. These terms were searched in the title, abstract, and keyword-related fields. A record was classified as within scope if a strong eye-tracking-related term was identified in the title or abstract, or if strong terms were identified simultaneously in the author-keyword (DE) and additional keyword (ID/KW) fields. The same rule set was applied uniformly to all 45,230 records without changing the original search strategy or manually modifying individual records.

The final rule-based screening identified 39,207 records as within scope and 6023 records (13.32% of the full corpus) as potentially off-topic. However, given the specificity of 76.7%, some genuinely out-of-scope records may remain among the 39,207 retained records. Therefore, the rule-based filtering reduces potential contamination but does not completely eliminate it. The difference between the 14.33% off-topic proportion observed in the manually validated sample and the 13.32% identified through full-corpus screening reflects the distinction between the sample-based estimate and the result obtained by applying the rule-based procedure to the complete dataset. When evaluated against the manual classification of the 300-record validation sample, the final rule achieved a sensitivity of 99.6%, specificity of 76.7%, accuracy of 96.3%, and precision of 96.2%, indicating a high level of overall agreement with the manual classification.

Using R, the principal bibliometric analyses were subsequently recomputed for the filtered corpus of 39,207 records. The results were compared with those obtained from the original corpus of 45,230 records to determine whether the exclusion of potentially off-topic records altered the main bibliometric patterns. The comparison focused on country-level production, countries by corresponding author, citation-based country rankings, institutional production, source productivity, and keyword occurrence. These indicators were selected to assess whether the potential contamination affected the principal rankings reported in the study and whether the headline findings remained robust after the exclusion of potentially off-topic records.

Table 5 presents the top 20 rankings for the original corpus (45,230 records) and the filtered corpus (39,207 records) across the six bibliometric indicators examined. Overall, the comparison suggests that the exclusion of potentially off-topic records did not substantially alter the broad ranking patterns, although differences were observed across the examined indicators. Country-level production and keyword occurrence showed the greatest stability, with the leading entities largely maintaining their relative positions.

The rankings of countries by corresponding author and the most cited countries also remained broadly consistent, despite some positional changes within the top 20. More noticeable changes were observed in institutional and source productivity, where several entities shifted their positions, and some were replaced within the top 20. These results suggest that the potential influence of off-topic records was more evident at the institutional and source levels than at the country and keyword levels. Overall, the main bibliometric patterns remained broadly consistent across the original and filtered datasets, supporting the general stability of the study findings while indicating some sensitivity in individual institutional and source rankings (Table 5).

## 4. Discussion and Conclusions

Although eye-tracking technology is recognized today as an important research method used across many disciplines, its origins date back approximately 150 years. Over this period, eye-tracking technologies have undergone a significant transformation, evolving from early approaches based on direct observation to high-precision digital systems, portable devices, and artificial intelligence-supported analysis methods. Along with technological advancements, the scope of eye-tracking methods has expanded, research output has increased significantly, and the field has evolved into a multidimensional research ecosystem to which an increasing number of disciplines contribute. This study evaluated the eye-tracking literature from a bibliometric perspective to reveal how this transformation has been reflected in scientific output.

By examining 45,230 publications indexed in the Scopus database and published between 1962 and 2025, this study provides a bibliometric assessment of eye-tracking research based on a substantially larger publication dataset than those used in several previous studies. The findings indicate that eye-tracking research has expanded substantially over the past six decades and has become increasingly connected to multiple disciplines and research contexts [35,51,52]. This finding is also consistent with previous studies reporting that eye-tracking technologies have become increasingly used across different disciplines in recent years [53,54]. Beyond describing publication and thematic patterns, the analysis shows that the share of eye-tracking publications in total Scopus output increased from 0.0134% in 2000 to 0.0963% in 2025. This indicates that the growth observed in the study was not solely attributable to the overall expansion of the Scopus database. In addition, the search sensitivity analysis demonstrates that changes in search terminology can substantially affect the retrieved corpus, highlighting the importance of terminology choices in interpreting bibliometric findings. The relatively low representation of non-English literature should also be considered when interpreting the findings, as the use of English search terms may have limited the retrieval of relevant studies published in other languages. Given that approximately 13–14% of the retrieved records may be off-topic, the absolute publication counts should be interpreted with caution. This is particularly relevant for institutional and source productivity, as some of the publications included in these counts may not be directly related to eye-tracking research. Therefore, the reported publication counts should be considered approximate upper-bound estimates rather than exact counts of eye-tracking publications.

Beyond its effect on corpus size, the search sensitivity analysis also has implications for the bibliometric representation of the field. The substantial differences in corpus size and composition obtained under alternative search strategies suggest that publication, institutional, source, and keyword patterns may vary depending on the terminology used to retrieve the literature. Thus, the bibliometric structure and apparent research trends of eye-tracking research should be interpreted in relation to the search terminology used to define the corpus. This finding highlights search-term selection as an important methodological consideration in bibliometric research on eye tracking.

One of the most important findings of the study is that the number of publications produced over the 58-year period from 1962 to 2019 was quite close to the number produced during the 2020–2025 period alone, suggesting that the field has undergone substantial growth in recent years [35,55]. This substantial growth may be associated with several concurrent developments, including the increasing accessibility of eye-tracking technologies alongside technological developments [53,56], advances in data-processing capacity [57], the growing use of artificial intelligence–supported analytical methods [24], and the increasing adoption of remote and technology-mediated research during the COVID-19 pandemic. These developments may have contributed to the acceleration of research activity after 2020 [58], although the present bibliometric analysis does not allow causal relationships to be established.

The distribution across subject areas suggests that eye-tracking research has evolved from a field primarily rooted in psychology and neuroscience into a multidisciplinary area connected with computer science, medicine, engineering, the social sciences, and other disciplines. The particularly high share of computer science is also consistent with previous studies reporting the increasing use of eye-tracking technologies in human–computer interaction, computer vision, and artificial intelligence applications [24,32].

The findings on countries, institutions, and collaboration networks suggest that eye-tracking research has developed within a strong global collaboration structure. The United States has maintained the highest level of scientific output for many years, while China has shown a notable increase in publication output in recent years. In addition, the international co-authorship rate and country-level collaboration networks suggest that knowledge production in the field increasingly involves international research collaborations and interdisciplinary teams. These collaboration patterns suggest that eye-tracking research is increasingly connecting researchers across different countries and disciplines, enabling the integration of diverse research expertise and perspectives. Such interconnectedness may indicate that the field is moving beyond a more specialized research focus toward a more integrated and collaborative research area, which may be considered a sign of its maturation. These findings are also broadly consistent with previous bibliometric studies of different thematic areas within eye-tracking research, which identified the United States, China, and Germany among the most productive countries [25,30,38,59]. However, productivity at the country and institutional levels should not be assessed solely on the basis of publication counts; differences in research capacity, disciplinary size, and publication and citation practices should also be taken into consideration. At the institutional level, publication counts should also be interpreted with caution because Scopus affiliation records may aggregate parent institutions, campuses, and affiliated units under a single institutional record. In particular, the University of California ranking may reflect the combined representation of multiple campuses and affiliated units within the system and therefore should not be interpreted as a direct comparison of individual campuses.

Keyword, source, and citation analyses, meanwhile, reveal the field’s theoretical and methodological transformation. Traditionally, research has focused on eye movements and visual attention. In recent years, however, it has increasingly addressed technology-oriented themes such as virtual reality [60,61], machine learning [57,62,63], deep learning [64,65], EEG, and human–computer interaction [24,32]. The growing prominence of machine learning and deep learning may be associated with the increasing use of computational approaches for processing and interpreting eye-movement data, while the emergence of virtual and augmented reality may reflect the expanding use of eye-tracking in immersive and interactive research environments. Similarly, the increasing visibility of EEG may indicate a growing interest in combining eye-movement measures with complementary neurophysiological data. Taken together, these trends suggest that eye-tracking research is increasingly being connected with computational, immersive, and neurophysiological approaches, extending its applications beyond the measurement of visual behavior alone. The thematic structure identified in this study is also consistent with recent review studies reporting the growing relevance of artificial intelligence and virtual reality applications in eye-tracking research [32,60].

Previous bibliometric studies have often focused on specific thematic areas, time periods, or more limited datasets. The present study extends this literature by examining the historical development, scientific output, collaboration patterns, and thematic structure of Scopus-indexed eye-tracking research between 1962 and 2025. More importantly, the normalized share analysis shows that the relative contribution of eye-tracking research to overall Scopus output increased over time, while the search sensitivity analysis demonstrates that alternative terminology can substantially change the size and composition of the retrieved corpus. Together, these findings extend the contribution of the study beyond descriptive bibliometric rankings by demonstrating both the relative growth of eye-tracking research within the broader Scopus literature and the sensitivity of its bibliometric representation to the terminology used to define the corpus.

## 5. Limitations and Future Research Directions

When evaluating the findings of this study, certain limitations must be taken into account. First, the research is based solely on publications indexed in the Scopus database. The primary reasons for this choice are Scopus’s broad interdisciplinary scope, its ability to export a large number of records, and the fact that the search strategy applied within the scope of this study yielded 45,230 publications. However, the use of a single database may have resulted in some studies indexed in other databases, such as Web of Science, PubMed, and IEEE Xplore, being excluded from the analysis. This limitation may be particularly relevant to older eye-tracking literature and to some psychology and neuroscience journals, for which coverage and indexing may vary across bibliographic databases. Therefore, the historical and disciplinary patterns identified in this study should be understood as reflecting the Scopus-indexed literature rather than the entirety of the eye-tracking literature. Future studies integrating and comparatively analyzing records obtained from different databases may provide a broader assessment of the scope and consistency of the findings.

Second, although no restriction was imposed on publication language, the search strategy was developed using only English keywords. Therefore, some publications that do not use English terms or that are indexed using equivalent expressions in other languages may not have been included in the dataset. In addition, the coverage and indexing characteristics of the Scopus database may also affect the representation of studies published in different languages to some extent. Furthermore, because the search strategy was limited to the specified keywords, some studies indexed using alternative terminology, specific device names, or other topic-specific keywords not included in the search query may also have been missed. Accordingly, potential language, coverage, and search strategy biases should be considered when interpreting the findings [66,67].

Third, the possibility that the search strategy included a certain proportion of out-of-scope records represents a limitation of the study. The validation results indicate that most of the dataset is relevant to eye-tracking research; however, the search strategy did not produce a dataset entirely free of out-of-scope records. Therefore, the potential influence of out-of-scope records should be considered when interpreting the bibliometric findings.

As a fourth limitation, although bibliometric analyses are effective in revealing the scientific production structure, collaboration networks, and thematic trends of a research field, they do not allow for a detailed assessment of the theoretical content and methodological characteristics of individual studies. Therefore, future research may complement bibliometric approaches with systematic reviews or content analysis.

As a fifth limitation, the broad scope of the keywords used in the data collection process may introduce certain constraints and uncertainties in the construction of the dataset. Although the search strategy aimed to identify publications related to eye-tracking research, it was observed that some studies included in the dataset did not directly address eye tracking as their research topic. For example, although some publications contained keywords related to eye tracking, it was determined that the studies primarily focused on behavioral tracking, motion tracking, or similar research areas. This situation can be considered a limitation inherent in large-scale bibliometric studies. In future research, the development of more detailed, subject-specific search strategies and the application of content-based validation processes could contribute to a more accurate analysis of the eye-tracking literature.

As a sixth limitation, in author productivity analyses, the matching of authors by surname and initials in Bibliometrix/Biblioshiny may result in different researchers being grouped under the same author label, particularly for researchers with common surnames such as Wang, Zhang, Li, and Liu. This limitation may affect the interpretation of author productivity findings; therefore, these results should be interpreted at the author-label level rather than at the level of individual researchers.

As a seventh limitation, differences in institutional naming and hierarchical structures within Scopus affiliation data should be taken into consideration when interpreting institutional productivity analyses. Different forms of institutional naming, the separate or combined representation of a parent institution and its affiliated units, and the inclusion of faculty or academic unit names as institutional entities may result in institutional publication output being represented differently. For example, the aggregation of multi-campus systems such as the University of California under a single institutional record may result in publications from different campuses being considered together. Similarly, the inclusion of academic units such as the College of Engineering in institutional lists, or the treatment of related institutional structures such as the University of London, University College London, and King’s College London, may result in overlapping counts in institutional productivity analyses. Therefore, findings on institutional productivity should be interpreted within the structure of the Scopus affiliation records. In future studies, standardizing institutional names and distinguishing institutional hierarchies may contribute to more precise analyses of institutional productivity.

Finally, another limitation of the study concerns the temporal coverage of the bibliometric dataset. Although the historical background was considered from 1879 onward, the earliest record in the bibliometric dataset dates from 1962. Therefore, the bibliometric trends identified in this study do not quantitatively represent the emergence and early development of eye-tracking research before 1962.

Future research may further examine the integration of eye-tracking technologies with emerging research areas such as artificial intelligence, augmented reality, virtual reality, and biometric data analytics. In addition, including patent documents in future bibliometric studies may contribute to a more comprehensive assessment of the development of the field, particularly by providing a broader perspective on technological developments in eye-tracking technologies.

## Figures and Tables

**Table 1:** Comparison of bibliometric studies conducted in the field of eye tracking.

Study	Database	Period	Number of Publications	Scope	Limitation
Ali et al. (2021) [32]	WoS	1992–2021	499	Vision Screening	Limited to the vision screening field.
Atabay & Güzeller (2021) [30].	Scopus	2013–2019	64	Tourism	Limited to the tourism field.
Muñoz Leiva et al. (2022) [27]	WoS	1992–2020	923	Marketing	Limited to the marketing field.
Kędras & Sobecki (2023) [33]	Scopus	2015–2020	93	Artificial Intelligence	Limited to artificial intelligence applications.
Shi (2024) [34]	WoS	1900–2022	1647	User Experience	Limited to the user experience field.
Dæhlen et al. (2024) [35]	WoS	2008–2023	26	Serious Games and Neurodevelopmental Disorders	Limited to serious games and neurodevelopmental disorders.
Dæhlen et al. (2023) [29]	WoS	2003–2023	28	Virtual Reality	Limited to virtual reality research.
Ruppenthal & Schweers (2024) [28]	WoS	2011–2023	118	Consumer Research	Limited to consumer research.
Prasanna Kumar et al. (2025) [36]	WoS	2005–2024	9773	All disciplines	Smaller dataset than the present study.
Lai et al. (2025) [25]	WoS	2001–2024	374	Educational Technology	Limited to educational technology research.
Siswono et al. (2025) [37]	Scopus	2013–2023	119	Mathematics Education	Limited to mathematics education.
Ayan (2025) [38]	WoS	2019–2023	8575	Distance/Online Learning and Machine Learning	Limited to distance/online learning and machine learning.
Umar & Mustafar (2025) [39]	Scopus	2015–2024	5825	Behavioural Sciences	Limited to behavioural sciences.
Kartal & Okyar (2025) [40]	WoS	2018–2022	245	Second Language	Limited to second language research.
Korkmaz (2026) [24].	WoS + Scopus	2020–2025	1033	Human–Computer Interaction	Limited to the 2020–2025 period.
Cherukat & Shabnam (2026) [26].	Scopus	1955–2025	6846	Visual Search	Limited to visual search research.
Baek et al. (2026) [41]	WoS	2000–2024	216	Advertising	Limited to advertising research.
Salgado-Fernández et al. (2022) [31]	WoS	1976–2021	4391	Academic Performance	Limited to eye movements and academic performance research.
This study	Scopus	1962–2025	45,230	All disciplines	Limited to Scopus-indexed publications and the selected English search terminology.

**Table 2:** Top 28 countries of corresponding authors ranked by publication output and international collaboration (SCP and MCP) (*n* = 45,230).

Country	No.	%	SCP	MCP	MCP (%)	Country	No.	%	SCP	MCP	MCP (%) ^1^
USA	6444	14.2	5343	1101	17.1	Poland	491	1.1	388	103	21.0
China	5016	11.1	3963	1053	21.0	Sweden	440	1.0	271	169	38.4
Germany	2764	6.1	2005	759	27.5	Belgium	338	0.7	186	152	45.0
UK	2251	5.0	1534	717	31.9	Austria	321	0.7	183	138	43.0
Japan	1256	2.8	1074	182	14.5	Finland	321	0.7	205	116	36.1
Canada	1212	2.7	880	332	27.4	Brazil	310	0.7	217	93	30.0
Netherlands	1014	2.2	639	375	37.0	Turkey	276	0.6	244	32	11.6
South Korea	946	2.1	798	148	15.6	Israel	267	0.6	165	102	38.2
Italy	871	1.9	596	275	31.6	Hong Kong	237	0.5	107	130	54.9
Australia	807	1.8	520	287	35.6	Norway	234	0.5	142	92	39.3
France	801	1.8	567	234	29.2	Denmark	219	0.5	102	117	53.4
Spain	704	1.6	476	228	32.4	Czech Republic	178	0.4	139	39	21.9
India	567	1.3	486	81	14.3	Portugal	177	0.4	106	71	40.1
Switzerland	525	1.2	298	227	43.2	Greece	148	0.3	109	39	26.4

No. = Number of Publications, ^1^ % = Percentage of Publications.

**Table 3:** Country-level scientific output and citation indicators.

Country	Country Affiliations	Proportion (%)	Total Citations (TC)	Average Article Citations	Country	Country Affiliations	Proportion (%)	Total Citations (TC)	Average Article Citations
USA	24,575	23.62	222,315	34.5	Spain	1836	1.76	13,176	18.7
Germany	13,373	12.85	65,382	23.7	Sweden	1770	1.70	11,825	26.9
United Kingdom	9336	8.97	67,475	30.0	Finland	1620	1.56	8777	27.3
China	7612	7.32	58,107	11.6	India	1285	1.23	4124	7.3
Japan	5092	4.89	13,991	11.1	Belgium	1169	1.12	9103	26.9
Canada	4308	4.14	31,050	25.6	Poland	1101	1.06	4834	9.8
Netherlands	3336	3.21	33,406	32.9	Austria	1064	1.02	4732	14.7
France	3261	3.13	16,600	20.7	Brazil	1053	1.01	3641	11.7
Australia	2930	2.82	19,031	23.6	Denmark	926	0.89	6734	30.7
Italy	2904	2.79	17,238	19.8	Israel	830	0.80	6836	25.6
Switzerland	2274	2.19	12,051	23.0	Turkey	786	0.76	3538	12.8
Korea	1961	1.88	11,864	12.5	Portugal	776	0.75	1794	10.1

Note: Country Affiliations and Proportion (%) are based on the Biblioshiny “Countries’ Scientific Production” analysis. Country Affiliations represent the country-level publication frequencies reported in this output. The denominator for Proportion (%) is *n* = 104,054, representing the total country-level publication frequency used for the calculation. Total Citations (TC) and Average Article Citations are obtained from the Biblioshiny “Most Cited Countries” analysis and use its corresponding-author-based counting unit. Accordingly, Average Article Citations is based on the corresponding-author publication count and is not calculated using the Country Affiliations values. The two sets of indicators therefore represent different counting units and are reported as provided by their respective Biblioshiny analyses.

**Table 4:** Thematic classification of eye-tracking research based on author keywords and associated countries.

Thematic Area	Most Frequently Used Author Keywords (Frequency)	Countries Most Frequently Associated with the Thematic Keywords
Eye Tracking Fundamentals	eye tracking (9719); eye-tracking (6492); eye movements (1764); eye movement (823); gaze tracking (704); gaze (569)	USA; Germany; China; United Kingdom; Canada
Visual Attention and Cognitive Processes	attention (1528); visual attention (1320); visual search (537); cognitive load (460); perception (307); visual perception (306); attentional bias (295); cognition (292); working memory (204); memory (199)	USA; Germany; United Kingdom; China; Canada
Artificial Intelligence and Computer Vision	machine learning (818); deep learning (507); computer vision (284); gaze estimation (267); artificial intelligence (232); prediction (224)	USA; China; Germany; India; United Kingdom
Human–Computer Interaction	human-computer interaction (403); usability (361); user experience (268); gaze interaction (179); human computer interaction (178)	USA; China; Germany; United Kingdom; India
Virtual and Extended Reality	virtual reality (1301); augmented reality (379)	USA; Germany; China; United Kingdom; Japan
Medicine and Neuroscience	EEG (571); autism (457); autism spectrum disorder (452); fmri (250); schizophrenia (235); electroencephalography (183)	USA; United Kingdom; China; Germany; Canada
Education, Reading and Learning	reading (566); children (246); learning (198); sentence processing (172)	USA; Germany; United Kingdom; Canada; Netherlands
Eye Movement Analysis	saccades (415); smooth pursuit (333); fixation (283); eye gaze (283); saccade (281); eye tracker (281); pupillometry (277)	USA; Germany; United Kingdom; Japan; China
Decision and Behavioural Research	emotion (305); social cognition (213); expertise (182); decision making (176); individual differences (173)	USA; United Kingdom; Germany; Canada; Netherlands

Note: Author Keywords frequencies represent their occurrences in the dataset. Countries were identified from the institutional affiliation (C1) information of publications containing the corresponding keywords. A publication may include affiliations from multiple countries and therefore contribute to more than one country count. For each thematic area, the five countries with the highest number of keyword–country associations are listed.

**Table 5:** Comparison of bibliometric rankings between the original and filtered corpora.

Analysis	Original Corpus (45,230)	Filtered Corpus (39,207)	Ranking Stability
Country production	USA; China; Germany; UK; Japan; Canada; Netherlands; France; Italy; Australia; Spain; South Korea; Switzerland; India; Sweden; Poland; Finland; Brazil; Austria; Belgium.	USA; China; Germany; UK; Japan; Canada; Netherlands; France; Italy; Australia; Spain; South Korea; India; Switzerland; Sweden; Poland; Finland; Brazil; Austria; Belgium.	Very high stability; only Switzerland and India changed positions.
Countries by corresponding author	USA; China; Germany; United Kingdom; Japan; Canada; Netherlands; Korea; Italy; Australia; France; Spain; India; Switzerland; Poland; Sweden; Belgium; Austria; Finland; Brazil.	USA; China; Germany; United Kingdom; Japan; Canada; Netherlands; Korea; Italy; France; Australia; Spain; India; Switzerland; Poland; Sweden; Finland; Austria; Brazil; Belgium.	High stability; minor changes in ranks 10–20.
Most cited countries	USA; United Kingdom; Germany; China; Netherlands; Canada; Australia; Italy; France; Japan; Spain; Switzerland; Korea; Sweden; Belgium; Finland; Israel; Denmark; Poland; Austria.	USA; United Kingdom; Germany; China; Netherlands; Canada; Australia; France; Italy; Sweden; Japan; Switzerland; Spain; Korea; Finland; Belgium; Denmark; Israel; Poland; Austria.	High stability; several changes in the middle ranks.
Institutional production	University of California; University of Toronto; Eberhard Karls Universität Tübingen; College of Engineering; University of London; Harvard Medical School; University College London; Shanghai Jiao Tong University; Universität Zürich; Universiteit Gent; The University of British Columbia; University of Illinois Urbana-Champaign; Ludwig-Maximilians-Universität München; Queen’s University; Beihang University; Vrije Universiteit Amsterdam; Tel Aviv University; King’s College London; Technische Universität München; Stanford University.	University of California; University of Toronto; Eberhard Karls Universität Tübingen; College of Engineering; University of London; Universiteit Gent; Universität Zürich; Shanghai Jiao Tong University; University of Illinois Urbana-Champaign; University College London; Queen’s University; The University of British Columbia; Ludwig-Maximilians-Universität München; Harvard Medical School; Universiteit Utrecht; Lunds Universitet; Technische Universität München; Uppsala Universitet; Vrije Universiteit Amsterdam; Tel Aviv University.	Moderate changes; leading institutions largely retained their positions.
Source productivity	Lecture Notes in Computer Science; PLOS ONE; Frontiers in Psychology; Vision Research; Proceedings of SPIE—The International Society for Optical Engineering; Sensors; ACM International Conference Proceeding Series; Conference on Human Factors in Computing Systems—Proceedings; Scientific Reports; Journal of Eye Movement Research; Journal of Vision; Experimental Brain Research; Journal of Neurophysiology; Advances in Intelligent Systems and Computing; Communications in Computer and Information Science; Proceedings of the Human Factors and Ergonomics Society; Investigative Ophthalmology and Visual Science; Cognition; Behavior Research Methods; Journal of Neuroscience.	Lecture Notes in Computer Science; Frontiers in Psychology; PLOS ONE; Proceedings of SPIE—The International Society for Optical Engineering; ACM International Conference Proceeding Series; Conference on Human Factors in Computing Systems—Proceedings; Journal of Eye Movement Research; Vision Research; Scientific Reports; Advances in Intelligent Systems and Computing; Sensors; Journal of Vision; Communications in Computer and Information Science; Proceedings of the Human Factors and Ergonomics Society; Journal of Neurophysiology; Behavior Research Methods; Experimental Brain Research; Cognition; IEEE Access; Applied Sciences (Switzerland).	Moderate-to-substantial changes; leading sources remained represented but several ranks shifted.
Keyword occurrence.	eye tracking; eye-tracking; eye movements; attention; visual attention; virtual reality; eye movement; machine learning; gaze tracking; eeg; gaze; reading; visual search; deep learning; cognitive load; autism; autism spectrum disorder; saccades; human-computer interaction; gaze behavior.	eye tracking; eye-tracking; eye movements; attention; visual attention; virtual reality; eye movement; machine learning; gaze tracking; reading; gaze; eeg; visual search; cognitive load; deep learning; autism spectrum disorder; saccades; autism; human-computer interaction; gaze behavior	Very high stability; only minor rank changes among lower-ranked terms.

## Data Availability

The Scopus dataset and analysis materials used in this study are openly available in Zenodo at https://doi.org/10.5281/zenodo.21904328 (accessed on 27 August 2026).

## Abbreviations

The following abbreviations are used in this manuscript:

WOS	Web of Science
MCP	Multiple Country Publications
SCP	Single Country Publications
EEG	Electroencephalography
EOG	Electrooculography
CR	Cited References
TC	Total Citations
ASD	Autism Spectrum Disorder
AI	Artificial Intelligence
HCI	Human–Computer Interaction
